# Chromatography Conditions Development by Design of Experiments for the Chemotype Differentiation of Four *Bauhinia* Species

**DOI:** 10.3389/fchem.2022.800729

**Published:** 2022-05-23

**Authors:** Amanda J. Aquino, Edenir R. Pereira-Filho, Regina V. Oliveira, Quezia B. Cass

**Affiliations:** ^1^ Separare—Núcleo de Pesquisa em Cromatografia, Departamento de Química, Universidade Federal de São Carlos, São Carlos, Brazil; ^2^ Grupo de Análise Instrumental Aplicada (GAIA), Departamento de Química, Universidade Federal de São Carlos, São Carlos, Brazil

**Keywords:** HRMS, hierarchical cluster analysis, *Bauhinia*, chemotype differentiation, dereplication, design of experiments

## Abstract

The extensive use of medicinal herbs to traditionally treat disease persists for generations, and scientific evidence on plant-derived extracts has indicated their numerous biological activities. The *Bauhinia*, popular known as cow’s paw (“pata de vaca”), with more than 60 native species, are extensively used in Brazilian popular medicine for the control of diabetes. Therefore, in 2009, *B. forficata*, *B. variegata* and/or *B. affinis* were included in the Brazilian National List of Medicinal Plants of Interest to SUS (RENISUS - Brazil). In this context, this work reports the results of the chemical differentiation of *B. forficata*, *B. variegata*, *B. longifolia*, and *B. affinis* using liquid chromatography coupled to high-resolution mass spectrometry and unsupervised chemometric tools. Chromatographic conditions were optimized by using the design of experiments (DoE) and chromatographic knowledge. Furthermore, the chemical profile of the studied species was analyzed by principal component analysis (PCA) and hierarchical cluster analysis that differentiated the four species of *Bauhinia*, and 55 compounds were also inferred by MS2 experiments, some of them for the first time in *B. affinis*. In this manner, this work provides important information that could be used in quality control, development of new pharmaceuticals, and food products based on *Bauhinia* leaves, as well as to explain ethnomedicinal properties, pharmacological and toxicological actions.

## Introduction

Widely used by the Brazilian population for the treatment and prevention of diabetes, plants of the genus *Bauhinia Linnaeus* (*Bauhinia L.*) are popularly known as cow’s paw (“pata de vaca”) or ox nail (“unha de boi”) because of its bilobed leaves (Henrique Domingos & Capellari Júnior, 2016).

The therapeutic properties of the cow’s paw leaf extracts are attributed to the presence of flavonoids that may act as hypoglycemic agents mainly in type 2 diabetes mellitus (de Souza et al., 2018). For instance, several biological studies have shown the antidiabetic activity attributed mainly to flavonoid glycosides, in particular kaempferitrin (kaempferol-3,7-di-O-α-L-rhamnopyranoside), a chemical marker reported for *B. forficata* leaves (da Cunha et al., 2010; De Sousa et al., 2004; Silva et al., 2002).

In Brazil, approximately more than 60 native species of *Bauhinia* are found (Cechinel Filho, 2009; Vaz et al., 2010). Teas of *Bauhinia* leaves or other plant-derived preparations have been widely used for the treatment of several illnesses, especially diabetes. Because of its high empirical use for medicinal purposes and the high interest in research with this plant species, the genus *Bauhinia* (*B. forficata*, *B. variegata* and/or *B. affinis*) was included in the RENISUS—National Relation of Medicinal Plants of Interest to the Unified Health System, Brazil, whose purpose is to foster research and development of monographs for quality control of herbal medicine (BRASIL, 2009).

The crescent commercial interest and therapeutic properties of herbal medicines undoubtedly require pharmacovigilance in the herbal medicinal products industry. Therefore, the monitoring of herbal medicines has become a major concern to both national health authorities and the public (Who, 2013). An effective regulation of plant-derived products must be established to improve their quality and avoid adverse reactions due to adulterations, misidentification of the medicinal plant species, inadequate quality control during the manufacturing, and/or poor-quality herbal preparations. For the protection of consumers and the development of relevant industries, authentication of medicinal plants represents a critical issue.

Usually, a medicinal herb contains hundreds of chemical constituents, so sophisticated separation and detection methods with high sensitivity and selectivity are required. Liquid chromatography coupled to high-resolution mass spectrometry (LC-HRMS) is widely used in the qualitative and quantitative analysis of natural product extracts since it provides a rapid and reliable picture of the plant’s chemical content. In the literature, several studies have been described for the characterization of chemical markers in natural products by liquid chromatography coupled to quadrupole time-of-flight mass spectrometry (LC-QTOF) for quality control, authentication/standardization, and differentiation of plant species (Xiao et al., 2013; Zhang et al., 2015; Chang et al., 2017; Xu et al., 2018).

Liquid chromatography (LC) is the analytical technique of choice for the analysis of diverse compounds in complex matrices. An effective analytical method development involves evaluating and optimizing different parameters to comply with the goals of the method. LC analytical methods are usually developed using the one-factor-at-a-time (OFAT) approach, in which one chromatographic parameter is varied in consecutive experiments until a satisfactory chromatographic resolution is obtained (Tome et al., 2019). The disadvantage of this strategy relies on the increased number of experiments and longer development time, especially when many parameters are affecting the separation. To circumvent this, the use of the design of experiments (DoE) analytical approaches, which systematically vary multiple key variables (e.g., pH, temperature, organic modifier, stationary phase, among others) simultaneously to obtain suitable experimental conditions with a minimum number of experiments (Tome et al., 2019; Thorsteinsdóttir & Thorsteinsdóttir, 2021). Screening and response-surface experimental designs allow the identification of significant factors, and the factor−response relationship is described by mathematical models, which can predict the optimal response. Multivariate combinations of key factors responsible for chromatographic performance promote reliable results for analytical method optimization and robustness.

Besides, new column technologies have furnished innovative stationary phase bonding chemistries and sub-2 μm fully porous and core-shell particles to mitigate some of the common problems associated with the traditional 5 μm and C18 associated with silica as solid support (Lopez et al., 2020). As a result, these new analytical opportunities provide faster LC analyzes with a gain of efficiency and chromatographic resolution. Moreover, significant advances have also been presented in the detection technologies, such as high-resolution mass spectrometers (HRMS) and nuclear magnetic resonance (NMR), making possible the comprehensive differentiation of isobaric or diastereomeric compounds (Wolfender et al., 2015).

This study describes the achievements of a high-efficiency LC method employing a three-step strategy for fingerprinting ethanolic extracts of *B. forficata* leaves, a specie recognized as a true cow’s-paw. In addition, the use of DoE for chromatographic separation and optimization, LC-HRMS analysis, and unsupervised statistical tools (principal component analysis, PCA and hierarchical cluster analysis, HCA) were carried out to classify four *Bauhinia* species (*B. forficata*, *B. variegata*, *B. longifolia*, and *B. affinis*) that are widely distributed in Brazil, being three of them already used as herbal medicines and *B. longifolia* was complementary selected to add to its phytochemistry database (Aquino et al., 2019). In this regard, 33 compounds were inferred as responsible for *Bauhinia* species chemical profiling, providing important information that could be used in quality control of these species, development of new pharmaceuticals and food products based on *Bauhinia* leaves, as well as to explain ethnomedicinal properties, pharmacological, and toxicological actions.

## Materials and Methods

### Chemicals

Methanol (HPLC grade) was used in all experiments and purchased from J.T. Baker (Philipsburg, United States). Formic acid (LC-MS grade) was acquired from Fluka (Buchs, Switzerland). Water was purified in a Milli-Q system (Millipore, São Paulo, Brazil).

### Plant Material

Leaves of *B. forficata* were obtained from the Multidisciplinary Research Center on Biological and Agricultural Chemistry—CPQBA—SP—Brazil (22.79° S, 47.11° W; 22,86° S, 47,07° W). The leaves of *B. longifolia* (22.87 °S, 47.07 °W; 22.86 °S, 47.08 °W) and *B. variegata* (22.98° S, 47.88° W; 22.70° S, 46.98° W; 22.70° S, 47.98° W) were obtained of the Agronomic Institute of Campinas—IAC—SP—Brazil. *B. forficata*, *B. variegata*, and *B. longifolia* were collected by V. A. P. e Carvalho (Carvalho, 2011). The leaves of *B. affinis* were obtained from the environmental preservation area—APA of the Ponta do Araçá—Porto Belo—Brazil—SC (27.13° S, 48.52° W) and collected by A. N. da Silva (Federal University of Santa Catarina). All species were identified by the Ph.D. researcher A. S. de F. Vaz, from the Research Institute of the Botanical Garden of Rio de Janeiro, Brazil. The specimen of *B. forficata* (755, 8333), *B. longifolia* (8325, 8330), and *B. variegata* (1112, 7655, 7658) was deposited in the herbarium of the Federal University of São Carlos (SPSC) and *B. affinis* (1884) was deposited in the herbarium of the botanical garden of Rio de Janeiro (JBRJ) and in the herbarium of the Botany Department of the Federal University of Santa Catarina (UFSC—FLOR61387). The collected samples have also been registered at SisGen Brazilian platform of Genetic Heritage and Associated Traditional Knowledge under the number A99450F. These vegetal samples were dried in a forced-air circulation drying oven set at 40°C for 7 days. For appropriated storage, the dried material was ground to a fine powder (60 Mesh).

### Sample Preparation

Powdered and dried samples (500 mg) were weighed and soaked with 5 ml of ethanol (LC grade) in conical tubes (50 ml) using an Ultraturrax^®^ homogenizer (IKA^®^, T18 basic model) set at speed 6 for 5 min. After grinding, the samples were centrifuged using a Jouan^®^ centrifuge (model, BR4i) for 10 min at 10,000 rpm and the supernatant (S1) was isolated. The S1 was evaporated using a Speed-Vac^®^ (Savant^®^, model SPD131DDA) set at 45°C overnight. The dried extracts obtained from the S1 fraction were labeled, weighed, and diluted to 50 mg/ml (D1) in MeOH:H_2_O (85:15; v/v).

The diluted extracts (D1) were cleaned up to eliminate chlorophylls. To this end, SPE cartridges (C18 end-capped, 100 mg, Varian^®^) with a volume capacity of 1 ml and a 20-port vacuum manifold system coupled to a Tecnal^®^ vacuum pump (model TE-058) were used. Initially, the SPE cartridge was activated with 2 ml of methanol (MeOH), equilibrated with 2 ml of MeOH:H_2_O (85:15; v/v), and then loaded with 500 μL of the D1. The eluate was collected, dried in a Speed-Vac^®^ set at 45°C overnight, and resuspended in MeOH:H_2_O (85:15; v/v) to yield a final concentration required for LC-HRMS analyzes. Before analysis, the samples were centrifuged at 9300 g for 10 min.

### Chromatographic Conditions

The UHPLC system (model Nexera^®^, Shimadzu) consisted of two quaternary LC-30AD pumps, a DGU-20A5R degasser, a SIL-30AC autoinjector, an SPD-M30A diode arrangement detector, a CTO-20AC furnace, a six-column selector valve, and a system Nexera Scouting Solution^®^ was used for the method development and sequence setup. All equipment units were controlled by a CBM 20A interface. The LabSolutions^®^ workstation software was used for operation of all modules and for data analysis and processing. The chromatographic columns selected were: Kinetex^®^ Biphenyl (100 mm × 2.1 mm x 2.7 µm) (Phenomenex, Torrance, CA, United States), Raptor^®^ Biphenyl (100 mm × 2.1 mm × 2.7 µm) (Restek, Bellefonte, PA, United States), Ascentis^®^ Express F5 (100 mm × 2.1 mm × 2.7 µm), and Ascentis^®^ Express C18 (100 mm × 2.1 mm × 2.7 µm) from Supelco (Bellefonte, PA, United States).

To calculate the number of chromatographic bands, the automatic integration parameters consisting of the same criteria were used for all samples to promote the highest number of bands in the chromatogram. The integration parameters were as follow: 1) width: 3 s; slope: 1,000 uV/min, drift: 0 uV/min, T.DBL (time required to double the peak width): 1,000 min; minimum area/weight: 1,000 counts; signal to noise ≥10, and the wavelength was fixed at 254 nm due to the solvent cutoff at different pH levels and the molar absorptivity of the compounds present in the plant matrices.

### Design of Experiments

The design of experiments (DoE), executed in three stages, was elaborated to produce the optimal chromatographic condition with a maximum number of chromatographic bands. Step 1 was carried out considering a full factorial design with all possible combination of high and low levels for all factors for three experimental critical factors: chemistry of the stationary phase, pH and organic modifier.

Initially, 51 experiments were carried out, of which 21 experiments were replicates run to estimate the confidence interval at the center point. Additionally, the gradient time and the column temperature were set at 10 min and 40°C, respectively. Next step (Step 2), the best chromatographic conditions obtained from Step 1 were selected and eight additional experiments were performed using a linear gradient of 30 min. The best condition of Step 2 was used in Step 3, in which the flow rate and the column temperature were modified to reduce the analysis time. For the calculation of the numbers of chromatographic bands, an automatic integration was applied to all chromatograms using the same criteria. The detection wavelength was set at 254 nm. The computational programs used for calculations were Excel^®^ (v.2019) and Matlab^®^ (v. R2013b 8.2.0.701).

### Liquid Chromatography Coupled to High-Resolution Mass Spectrometry Analysis

For the principal component analysis of 4 *Bauhinia ssp*, a liquid chromatography separation was obtained with a Kinetex^®^ Biphenyl column (100 mm × 2.1 mm; 2.7 μm) (Phenomenex, Torrance, CA, United States) equipped with a guard-column. The solvents used were 100 µmol/L of formic acid in water (solvent A) and 100 µmol/L of formic acid in methanol (solvent B) as mobile phase, flow rate of 0.7 ml/min, and temperature set at 50°C. Gradient elution of 25 min run time was carried out using the following steps: 0 min, 5% B; 0–20 min, 5%–70% B; 20.1–25 min, 100% B for column cleaning and a conditioning time of 5 min with 5% B. The injection volume was 0.5 μl (sample concentration: 30 mg/ml).

The UHPLC (Nexera, Shimadzu) system, previously described, was coupled to an Impact HD QTOF mass spectrometer (quadrupole time-of-flight mass spectrometry—Bruker Daltonics, Bremen, Germany) equipped with an electrospray (ESI) interface operating in the positive or negative ion mode. Negative ionization mode was chosen for promoting better sensitivity for a higher number of metabolites under the analytical conditions tested (Liigand et al., 2017). A flow splitter was placed between the LC column exit and the MS, and the flow rate arriving at the MS was set at 118 µl/min. The optimal mass spectrometer parameters were set as follows: capillary voltage, 4000 V; endplate offset, 500 V; nebulizer, 1 bar; dry heater temperature, 250°C; dry gas flow, 8 L/min; collision cell energy, 5 eV, and *full-MS* scan range, *m/z* 50–1500.

For characterization of the secondary metabolites, the mass spectrometer was programmed to perform acquisition in Auto MS/MS mode (number of precursors 5) and MS/MS experiments were performed using different collision energies of 10, 20, 25, 30, 35, 40, 45, or 50 eV for all *m/z* interval range. Data acquisition and processing were performed using Data Analysis^®^ and Profile Analysis^®^ v.2.1 software (Bruker Daltonics GmbH, Bremen, Germany). Structural chemical characterization was established based on MS/MS fragmentation pattern, accurate mass, and comparison to spectral library (CompoundCrawler™, MetFrag, and SmartFormula^™^ from Bruker Daltonics GmbH, Bremen, Germany) and public databases structure search based on formula (http://mona.fiehnlab.ucdavis.edu/; http://www.massbank.eu/; https://metlin.scripps.edu) to enable the creation of annotations at increasing levels of specificity and confidence. Molecular formulas of the identified compounds were calculated using an accurate mass error <10 ppm. The identified compounds were searched in the literature to check if they were present in the *Bauhinia* ssp and examined using the HMDB database (http://www.hmdb.ca/) to learn if they were in the plant kingdom and/or in the Fabaceae family, as previously published for *B. longifolia* (Aquino et al., 2019).

## Results and Discussion

### Analytical Development by Design of Experiments

The analytical method development and optimization were performed through a design of experiment (DoE) study by modifying simultaneously multiple critical method parameters (CMPs), which can affect critical method attributes (CMAs) such as resolution (Rs), peak tailing (T), and selectivity (*α*) (Lloyd R. Snyder & Dolan, 2013).

The plant extract chosen for the LC method development was the ethanolic extract of *B. forficata* leaves. The reverse mode of elution was used due to the properties of the analytes present in plant matrices being of medium polarity to non-polar, and for the same reasons, the use of ionic paring was not considered as a variable. The orthogonality values for the pre-selected stationary phases were obtained from the results of *Fs* described on the website of the American Pharmacopoeia, which is based on the theory of hydrophobic subtraction developed by Snyder and collaborators (USP, 2005; Snyder & Dolan, 2013; Cass, 2015; Lloyd R.).

Thus, the Kinetex^®^ biphenyl (*Fs* = 0) column was compared to three other columns: the Ascentis^®^ Express F5, Ascentis^®^ Express C18, and the Raptor^®^ biphenyl, being considered orthogonal only to the Ascentis^®^ Express F5 and the Ascentis^®^ Express C18 columns with an *Fs* > 10 (USP, 2005).

The organic modifier was selected according to Snyder-Rohrschneider’s selectivity triangle which considers the different chemical interactions of the organic modifiers—acidity, basicity, and dipole interactions (L. R. Snyder et al., 1993). In this work, only the use of methanol and acetonitrile were investigated because of the incompatibility of tetrahydrofuran with modern LC equipment.

Initially, a full factorial design was carried out (DoE—Step 1), in which all variables, their interactions, and levels were considered in the calculations. This design allows the screening of a high number of factors with fewer experiments and can also be used for robustness or ruggedness testing (Thorsteinsdóttir & Thorsteinsdóttir, 2021). To meet this end, three critical method parameters that mainly affect chromatographic selectivity were considered: 1) chemistry of the stationary phase (column); 2) type of organic modifier, and 3) pH values. All CMPs and levels are shown in Table 1.

**TABLE 1:** Critical method parameters (CMPs) and levels used in the development of the chromatographic conditions in Step 1.

Selectivity factor (*F*_*s*_)	Column	Column levels	Organic modifiers (OM)	OM levels	pH effective		pH levels
0	Kinetex^®^ biphenyl (100 mm × 2.1 mm); 2.7 µm	−1	Methanol (MeOH)	1	3.7	Ammonium formate 10 mM	−1
9	Raptor^®^ Biphenyl (100 mm × 2.1 mm); 2.7 µm	−0.3791	Acetonitrile (MeCN)	−1	4.2	Formic acid 200 uM	−0.7297
20	Ascentis^®^ Express F5 (100 mm × 2.1 mm); 2.7 µm	0.3791			5.3	Ammonium acetate 10 mM	−0.1351
29	Ascentis^®^ Express C18 (100 mm × 2.1 mm); 2.7 µm	1			7.4	Ammonium bicarbonate 10 mM	1

Thus, three main steps were considered for the development and optimization of the chromatographic separation: 1) screening of the variables that most impact the selectivity—stationary phase, pH, and organic modifier; 2) fine adjustment of peak capacity by using the best conditions from Step 1 and applying a different gradient time; 3) optimization of analysis time by keeping the chromatographic resolution without losing selectivity. To this end, higher flow rate and temperature were considered.

The results encoded variables and levels of DoE—Step 1 are shown in Supplementary Table S1, and the results of the analysis of variance (ANOVA) are shown in Supplementary Table S2. It was verified that the regression model selected was an adequate fit to the data using an *F* test, where the mean square regression (MSR) and regression mean residual (MS_residual_) were statistically significant, with an *F1*_calculated_ > *F1*_critical_ at 95% confidence level. These results produced a mathematical model, where a second-order equation (Eq. 1) describes the results obtained from DoE—Step 1:

$$y=67.86-12.14C-6.22pH+4.29O+10.63C^{2}-11.99pH^{2}\left(\pm 6.9\right)\left(\pm 3.6\right)\left(\pm 3.7\right)\left(\pm 2.8\right)\left(\pm 6.1\right)\left(\pm 7.7\right)$$ (1)

Where y is the dependent variable that accounts for the number of chromatographic bands, C is the column type, pH is the acidity/basicity value of the aqueous mobile phase, and O is the type of organic modifier used.

**FIGURE 1:**
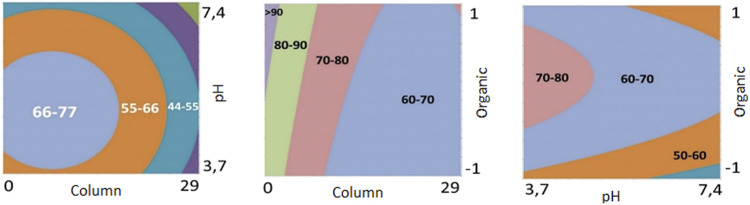
Contour plots obtained in DoE—Step 1 of the experimental design.

The results obtained for DoE—Step 1 showed that the Kinetex^®^ Biphenyl column promoted a higher number of chromatographic bands when used at lower pH levels. Also, the contour plots (Figure 1) for the Kinetex^®^ biphenyl column demonstrate these findings, and it was the chromatographic column selected for further experiments. Therefore, different levels for pH (3–5), organic modifier (methanol and acetonitrile), and a linear gradient time of 10 and 30 min, Table 2, were used as the next critical factors during the DoE—Step 2 of the chromatographic optimization. Therefore, eight more analyzes were performed, with 2 replicates, using the Kinetex^®^ biphenyl column, temperature 40°C, and flow rate of 0.5 ml/min.

**TABLE 2:** Variables used in DoE—Step 2.

Time (min)	Organic modifier	Aqueous pH	
30	MeOH	3.7	Ammonium formate 10 mM
10	MeCN	4.2	Formic acid 200uM
		5.3	Ammonium acetate 10 mM

*Fixed condition: Kinetex^®^ biphenyl column, temperature 40°C, and flow rate 0.5 ml/min.

The results obtained from DoE—Step 2 (Table 2) were also analyzed using ANOVA and Student’s t-test (with 4 degrees of freedom), and it was verified that the pH value showed no significant effect in the studied value range (pH 3–5), then it was fixed in pH 4.2 using 200 μM of formic acid in the mobile phase for further experiments. The results obtained when using pH 4.2 promoted higher signal intensities for the detected ions in the negative ionization mode.

Because replicated experiments have been carried out (*n* = 2), the residual component from the total variation was assembled from the lack of fit (LoF) component and a component due to pure experimental error (Tome et al., 2019). In our results, the square mean of lack of adjustment (MS_LoF_) and the square mean of the pure error (MS_PE_) was statistically equal with an *F2*_calculated_ < *F2*_critical_, indicating that the model did not lack of fit. In addition, the mean square regression (MSR) and regression mean residual (MS_residual_) were statistically different. Therefore, the model was statistically significant with *F1*_calculated_ > *F1*_critical_ and the coefficient of determination (R^2^) equal to 0.97, also indicating an adequate adjustment of the model to the observed responses at a confidence level of 95%. For a full list of the parameters used from the DoE—Step 2 refer to Supplementary Table S3.

**FIGURE 2:**
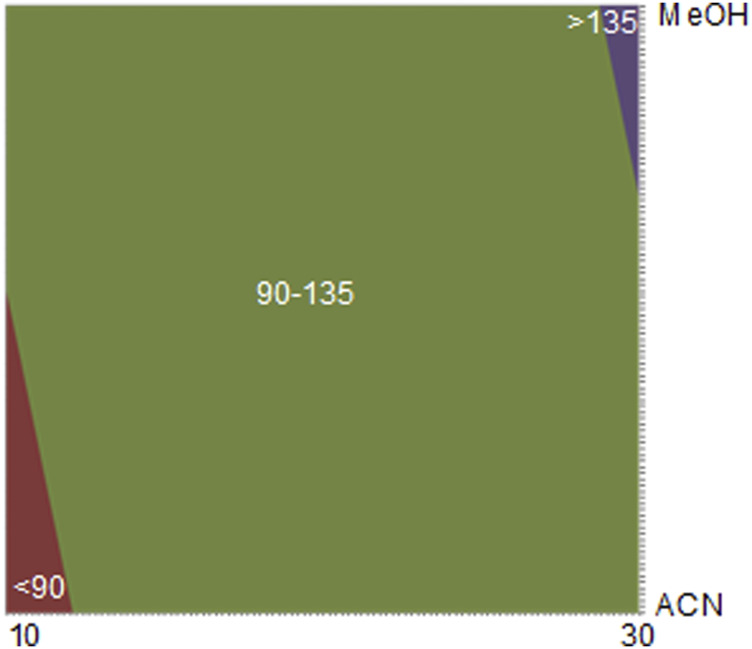
Contour plot obtained in DoE—Step 2. [* The values in the contour chart refer to the number of chromatographic bands. ** Fixed condition: Kinetex^®^ Biphenyl column, pH 4.2 (formic acid 200 μM), temperature 40°C, and flow rate 0.5 ml/min X axis (time—minutes) and Y axis organic modifier.

After applying the Student’s *t*-test (with 12 degrees of freedom), both gradient slope (I) and organic modifier (O) were statistically significant. Thus, with Eq. 2, the contour plot was constructed and displayed in Figure 2, showing that when using longer analysis time and methanol as an organic modifier, the highest number of resolved chromatographic bands is obtained.

y=111.56+21.85I+4.32O(±5.4)(±3.4)(±3.6) (2)

The Kinetex^®^ biphenyl stationary phase and methanol as the organic modifier showed the highest resolution for a larger number of chromatographic bands. This could be justified due to the presence of many phenolic compounds in these species (Aquino et al., 2019).

**FIGURE 3:**
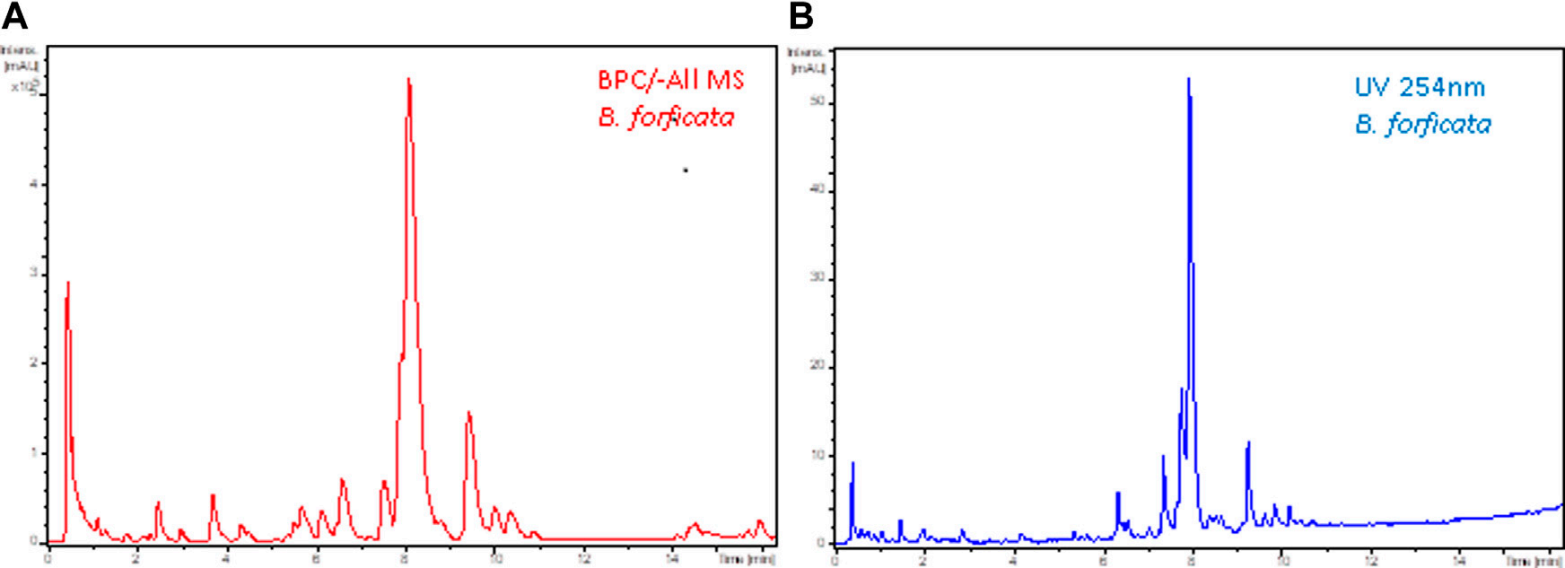
Final chromatogram after DoE—Step 2 of leaf ethanolic extract *Bauhinia forficata* **(A)** base ion chromatogram in the negative ionization mode (-All/MS), and **(B)** by UV at 254 nm. Chromatographic conditions: Kinetex^®^ Biphenyl column, flow rate 0.7 ml/min, temperature 50°C. Gradient: 5%–70% B.

In DoE—Step 3, chromatographic adjustments were made to decrease the analysis time but to preserve the same number of chromatographic bands and resolution. The flow rate of 0.5 ml/min and temperature 40°C were increased to 0.7 ml/min and 50°C, respectively. The final chromatographic condition in the LC-HRMS and LC-UV is shown in Figure 3.

### Dereplication of the Four *Bauhinia* Species

**FIGURE 4:**
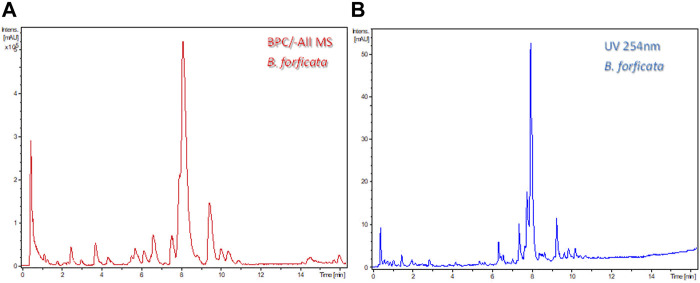
Survey view (SV) and base peak chromatograms (BPC) in negative ionization mode (-All/MS) for *B. forficata* (1a and 1b), *B. variegata* (2a and 2b), *B. affinis* (3a and 3b), and *B. longifolia* (4a and 4b).

The previously developed chromatographic conditions were used for the analysis of the ethanolic extracts of *B. forficata*, *B. variegata*, *B. longifolia*, and *B. affinis* by LC-HRMS to identify the compounds that could differentiate these species. The obtained chromatograms and MS survey spectra (SV) are shown in Figure 4. The MS survey view displays the density data of the selected chromatogram analysis by recording the retention time and the *m/z* value. The intensity values that correspond to the retention time—*m/z* pairs are expressed by a color code, where darker blue means more intense MS signals.

The data evaluation of the MS data using statistical techniques was carried out using the Profile Analysis software (Brucker Daltonics), which calculate the molecular features by using numerical values obtained from the grouping of ions by retention time (t_R_) and isotope pattern, and by unifying *m/z* of the same ion (different charged ions, adducts or clusters). This preprocessing bucketing method performs a data reduction by grouping highly similar spectra, after which each bucket can be represented by a single consensus spectrum (Bittremieux et al., 2021). Moreover, ion intensities were normalized by the sum of the values of the buckets, and these were tabulated. As a result, 541 buckets (S/N 5^a^) were obtained from the selection parameters described in Supplementary Table S4.

The explained variance plot, obtained by plotting the percentage of the variance explained by the extracted PC, was used to identify the ideal number of principal components (PCs) and the resulting curve profile used to determine the cut-off point by decreasing the slope observed, which in this case was the third principal component. Three PCs were selected, which explained 93.7% of the total data variation. The principal component 1 (PC1) was responsible for 61.2% of the original data variance, whereas PC2 and PC3 corresponded to 25.3% and 7.2%, respectively.

The obtained MS data were evaluated to further contribute to the phytochemical studies of the genus *Bauhinia*. The dereplication of the compounds was feasible due to manually comparison of the exact masses, MS/MS fragmentation patterns, and isotopic contribution pattern with those data reported in the literature or already deposited in spectral libraries online (http://mona.fiehnlab.ucdavis.edu/; http://www.massbank.eu/; https://metlin.scripps.edu), and data acquired in LC-MS systems equipped with an ESI source and fragmentation through collision-induced dissociation (CID). Furthermore, the identified compounds were searched in the literature to verify if they were present in the *Bauhinia ssp*, consulted in the HMDB database (http://www.hmdb.ca/) to know if they were present in the plant kingdom and/or in the Fabaceae family. The compounds were inferred by excluding those ions which were fragment ions of the precursor, adducts, and clusters at the same retention time. Supplementary Table S5 illustrates the 55 compounds inferred by dereplication and listed in numerical order according to their retention time. The LC-HRMS spectra data is also presented in the Supplementary Material, Section 1. Supplementary Table S6 shows the presence of the 55 compounds in the ethanolic extracts (*n* = 2) of *B. forficata*, *B. variegata*, *B. longifolia*, and *B. affinis* leaves, at the experimental conditions evaluated for sample extraction and analysis.

### Differentiation of the *Bauhinia* Species Using Chemometric Tools

**FIGURE 5:**
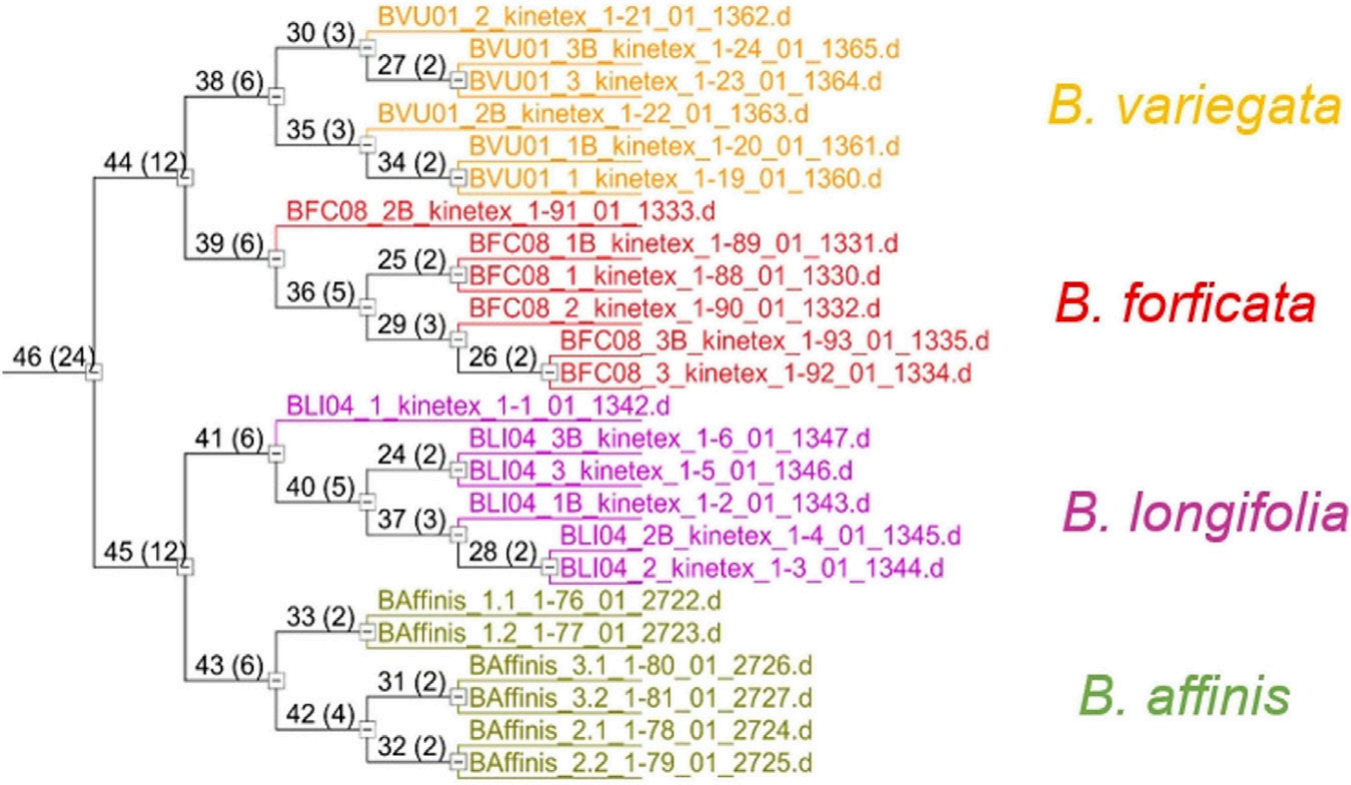
Hierarchical clustering analysis of the 4 studied *Bauhinia* species.

The inferred compounds from the molecular characteristics table (Supplementary Table S5) were used for species classification and differentiation using the unsupervised pattern recognition, cluster analysis (HCA), and principal component analysis (PCA) methods. In the dendrogram obtained with the HCA (Figure 5) it was possible to see four large groups formed based on the existing similarities in relation to the analyzed variables, indicating clear differences between the *Bauhinia* species.

To get a better understanding of the compounds that differentiate the species, PCA was applied. The explained variance plot, obtained by plotting the percentage of the variance explained by the extracted PC, was used to identify the ideal number of principal components (PCs) and the resulting curve profile used to determine the cut-off point by decreasing the slope observed, which in this case was the third principal component. Three PCs were selected, which explained 98.7% of the total data variation. The principal component 1 (PC1) was responsible for 80.0% of the original data variance, whereas PC2 and PC3 corresponded to 12.3% and 6.0%, respectively (Supplementary Figure S1).

**FIGURE 6:**
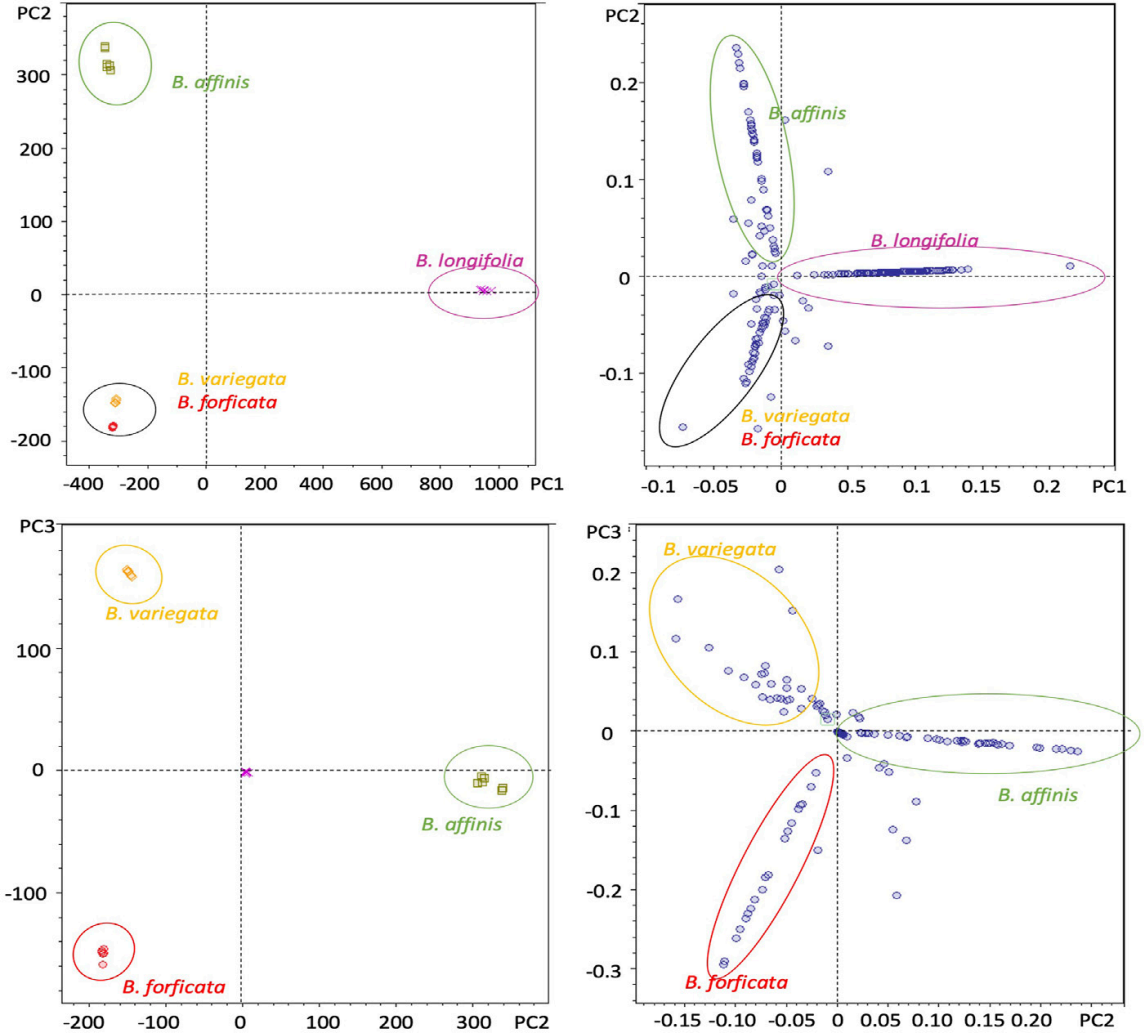
Principal component analysis (PCA) of *B. forficata, B. variegata, B. longifolia*, and *B. affinis*. Upper: PC1 vs. PC2 (Score graphs). Bottom: PC2 vs. PC3 (loading graphs).

The samples of each species were grouped in scores graphs (PC1 vs. PC2), Figure 6A, with differentiation between the species *Bauhinia longifolia and Bauhinia affinis*, while grouping *Bauhinia forficata* and *Bauhinia variegata*. In the loading graphs (PC2 vs. PC3) (Figure 6B) it was possible to verify the separation of *Bauhinia forficata*, *Bauhinia variegate*, and *Bauhinia a*ffinis.

### Secondary Metabolites Chemical Characterization by Liquid Chromatography Coupled to High-Resolution Mass Spectrometry

The compounds 2, 9–12, 15–18, 25, 26, 28, 31, 33, 41,42, 44, 46, 50–52, and 55 listed in Supplementary Table S5 have been previously inferred by the authors for *Bauhinia longifolia* extract (Aquino et al., 2019), while the discussion and spectra of the other metabolites annotated in this article are disclosed in the Supplementary Material.

Additionally, it was not possible to verify the presence of kaempferitrin, the alleged chemical marker of *Bauhinia forficata*. For that, we searched the data using the ion extracted chromatograms (EIC), considering the deprotonated molecular ion *m/z* 577.1552 (M-H)^−^, adducts such as [(M-AF)^−^, (M-H_2_O-H)^−^, and (2M + AF-H)^−^], and their respective fragment ions. Our data corroborate with those previously published by Ferreres et al. (2012), in which kaempferitrin was not detected in *B. forficata Link subsp. Pruinosa* (*Vogel*) *Fortunato & Wunderlin*.

Bauhiniastatin 2 is present in all species studied in this work. This compound has been reported in *B. purpurea* and its medicinal properties are related to anticancer activities (Pettit et al., 2006), which demonstrates the potential of *Bauhinia* species for this purpose.

In all species studied, a wide variety of phenolic compounds were identified, however, to date, no results of biological and toxicological activities have been reported for the plant-derived extractions of *B. affinis* and very few for *B. longifolia*.

## Conclusion

The use of DoE for the LC method development using extracts of *Bauhinia forficate* as a model, allowed a rapid and efficient method development since multiple CMPs were simultaneously evaluated. The DoE approach also provided an increased selectivity for a complex mixture of compounds in a natural product matrix. The obtained chromatograms promoted a satisfactory number of resolved bands and the LC coupling to HRMS and further data analysis by principal component analysis allowed the dereplication of a series of compounds and differentiation of the four studied *Bauhinia* species.

Bauhiniastin 2 is herein reported for the four species, as well as a wide variety of phenolic compounds providing important information to the herbal medicine industry; however, it is important to highlight, that up to date, no results of biological and/or toxicological activities have been reported for *B. affinis* and very scarce results are presented for *B. longifolia*. Thus, further studies are needed for their safe plant medicinal use.

The presented work demonstrated that the use of DoE for method development of plant-derived extracts and analytical approaches by LC-HRMS associated with unsupervised chemometric methods is a powerful tool for the chemical differentiation of plant species and can be further used in plant authentication, especially in Brazil where there is a growth in the use of herbal medicines for the Brazilian population through the Unique Health System (SUS).

## Data Availability

The original contributions presented in the study are included in the article/Supplementary Material, further inquiries can be directed to the corresponding authors.
