# Supplementary material for: Chromatography Conditions Development by Design of Experiments for the Chemotype Differentiation of Four Bauhinia Species
Source: Front Chem. 2022 May 23;10:800729. doi: 10.3389/fchem.2022.800729 (PMC9169091; doi:10.3389/fchem.2022.800729)
Supplement: Supplementary file 1 [file DataSheet1.pdf]

Supplementary Material

Chromatography Conditions Development by Design of Experiments for the Chemotype Differentiation of Four Bauhinia Species

Amanda J. Aquino[1], Edenir R. Pereira-Filho[2], Regina V. Oliveira[1†], Quezia B. Cass[1†]

[1] *Separare* – Núcleo de Pesquisa em Cromatografia, Departamento de Química, Universidade Federal de São Carlos, São Carlos, SP, Brazil
[2] Grupo de Análise Instrumental Aplicada (GAIA), Departamento de Química, Universidade Federal de São Carlos, São Carlos, SP, Brazil

[†] These authors have contributed equally to this work and share last authorship
Correspondence:

R.V. Oliveira oliveirarv@ufscar.br

Q.B. Cass qcass@ufscar.br

1 Supplementary Figures

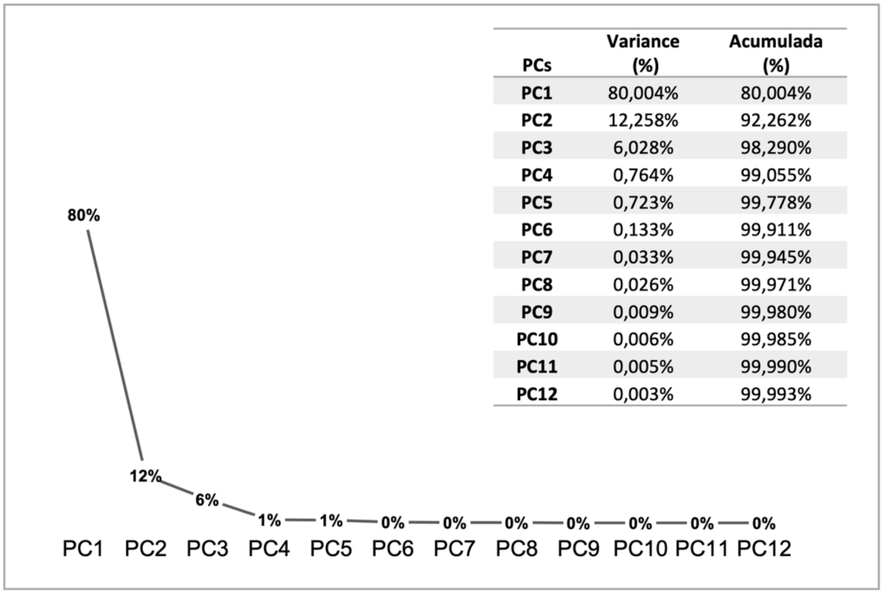

PCs	Variance (%)	Acumulada (%)
PC1	80,004%	80,004%
PC2	12,258%	92,262%
PC3	6,028%	98,290%
PC4	0,764%	99,055%
PC5	0,723%	99,778%
PC6	0,133%	99,911%
PC7	0,033%	99,945%
PC8	0,026%	99,971%
PC9	0,009%	99,980%
PC10	0,006%	99,985%
PC11	0,005%	99,990%
PC12	0,003%	99,993%

Figure S1. Explained variance chart (%) for choosing the number of main components used in the PCA data processing.

2 Supplementary Tables

Table 1S. Results obtained from DoE – Step 1 showing number of chromatographic bands for tested column

Column	Normalized Levels (columns)	pH	Normalized Levels (pH)	Organic Modifier	Levels	Responses (nº of chromatographic bands)
Raptor® Biphenyl	-0.3791	5.3	-0.1351	MeCN	-1	72
Raptor® Biphenyl	-0.3791	4.2	-0.7298	MeCN	-1	74
Raptor® Biphenyl	-0.3791	7.4	1	MeCN	-1	48
Raptor® Biphenyl	-0.3791	7.4	1	MeCN	-1	47
Raptor® Biphenyl	-0.3791	3.7	-1	MeCN	-1	65
Raptor® Biphenyl	-0.3791	3.7	-1	MeCN	-1	58
Raptor® Biphenyl	-0.3791	5.3	-0.1351	MeOH	1	84
Raptor® Biphenyl	-0.3791	4.2	-0.7298	MeOH	1	81
Raptor® Biphenyl	-0.3791	4.2	-0.7298	MeOH	1	80
Raptor® Biphenyl	-0.3791	4.2	-0.7298	MeOH	1	82
Raptor® Biphenyl	-0.3791	4.2	-0.7298	MeOH	1	84
Raptor® Biphenyl	-0.3791	4.2	-0.7298	MeOH	1	87
Raptor® Biphenyl	-0.3791	4.2	-0.7298	MeOH	1	85
Raptor® Biphenyl	-0.3791	4.2	-0.7298	MeOH	1	85
Raptor® Biphenyl	-0.3791	7.4	1	MeOH	1	64
Raptor® Biphenyl	-0.3791	7.4	1	MeOH	1	57
Raptor® Biphenyl	-0.3791	3.7	-1	MeOH	1	77
Raptor® Biphenyl	-0.3791	3.7	-1	MeOH	1	67
Kinetex® Biphenyl	-1	5.3	-0.1351	MeCN	-1	82
Kinetex® Biphenyl	-1	4.2	-0.7298	MeCN	-1	90
Kinetex® Biphenyl	-1	7.4	1	MeCN	-1	77
Kinetex® Biphenyl	-1	3.7	-1	MeCN	-1	76
Kinetex® Biphenyl	-1	5.3	-0.1351	MeOH	1	89
Kinetex® Biphenyl	-1	4.2	-0.7298	MeOH	1	90
Kinetex® Biphenyl	-1	4.2	-0.7298	MeOH	1	92
Kinetex® Biphenyl	-1	4.2	-0.7298	MeOH	1	94
Kinetex® Biphenyl	-1	7.4	1	MeOH	1	71
Kinetex® Biphenyl	-1	7.4	1	MeOH	1	69
Kinetex® Biphenyl	-1	3.7	-1	MeOH	1	89
Ascentis® Express F5	0.3791	5.3	-0.1351	MeCN	-1	51
Ascentis® Express F5	0.3791	3.7	-1	MeCN	-1	43
Ascentis® Express F5	0.3791	5.3	-0.1351	MeOH	1	47
Ascentis® Express F5	0.3791	4.2	-0.7298	MeOH	1	80
Ascentis® Express F5	0.3791	4.2	-0.7298	MeOH	1	77
Ascentis® Express F5	0.3791	4.2	-0.7298	MeOH	1	75
Ascentis® Express F5	0.3791	4.2	-0.7298	MeOH	1	78
Ascentis® Express F5	0.3791	4.2	-0.7298	MeOH	1	47
Ascentis® Express F5	0.3791	7.4	1	MeOH	1	49
Ascentis® Express F5	0.3791	3.7	-1	MeOH	1	40
Ascentis® Express C18	1	5.3	-0.1351	MeCN	-1	68
Ascentis® Express C18	1	4.2	-0.7298	MeCN	-1	67
Ascentis® Express C18	1	7.4	1	MeCN	-1	55
Ascentis® Express C18	1	3.7	-1	MeCN	-1	55
Ascentis® Express C18	1	5.3	-0.1351	MeOH	1	62
Ascentis® Express C18	1	4.2	-0.7298	MeOH	1	66
Ascentis® Express C18	1	4.2	-0.7298	MeOH	1	69
Ascentis® Express C18	1	4.2	-0.7298	MeOH	1	70
Ascentis® Express C18	1	4.2	-0.7298	MeOH	1	71
Ascentis® Express C18	1	4.2	-0.7298	MeOH	1	70
Ascentis® Express C18	1	7.4	1	MeOH	1	61
Ascentis® Express C18	1	3.7	-1	MeOH	1	60

Table S2. Analysis of variance from the DoE - Step 1.

Source of variation	Quadratic Sum	Degree of freedom	Quadratic mean	Fcal (95%)	Ftab (95%)	R^2	
Regression (R)	7045.81	9	782.87	10.09	2.12	0.69	F1
Residue (r)	3180.23	41	77.57				
Pure error (EP)	175.01	19	9.21	14.83	2.13		
Lack of adjustment (Faj)	3005.22	22	136.60				
Total	10226	50	204.52				

Table S3. Analysis of variance from DoE - Step 2

Source of variation	Quadratic Sum	Degree of freedom	Quadratic mean	Fcal (95%)	Ftab (95%)	R^2	
Regression (R)	7799.53	4	1949.89	81.43	3.36	0.97	F1
Residue (r)	263.41	11	23.95	0			
Pure Error (EP)	24	4	6	5.70	6.09		F2
Lack of adjustment (Faj)	239.41	7	34.21	0			
Total	8062.94	15	537.53	0			

Table S4. Parameters for calculations of molecular characteristics by principal component analysis (PCA)

Treatment	Parameter	Limiar value
By analysis (samples) and calculation of buckets	Signal to Noise Ratio (S/N)	5[a] or 15[b]
	Correlation Coefficient Threshold	0.7
	Minimum mass spectrum signal width*	15
	Advanced bucket	0.4 min e 1mDa
	Normalization	Sum of bucket values in analyzes
	Bucket filter	>=6 buckets within the *Bauhinia* group
		Replace lost bucket values in the calculations by means of the *Bauhinia* group
	Smoothing width	7
	Adducts and clusters	$[M-H]^-$; $[M+HCOOH-H]^-$; $[M+CH_3COOH-H]^-$; $[2M-H]^-$; $[2M+HCOOH-H]^-$; $[2M+CH_3COOH-H]^-$;$[3M-H]^-$.
Variables (*buckets*)	PCA e HCA	Variance
Calculation of principal component analysis	% Variance	

Table S5. LC-HRMS data of the 55 inferred compounds identified based on the data of Brucker spectral libraries

Compound ID	Compound name	Rt (min)	*m/z* Experimental $[M-H]^-$	Error (ppm)	Molecular formula $[M-H]^-$	Collision Energy (eV)	Fragment ions (%)
1	Hexose-hexose	0.5	341.1093	-1.1	$C_{12}H_{21}O_{11}$	20	179.0563(100); 161.0461(54.1)
2	Gallic acid	0.6	169.0140	1.5	$C_7H_5O_5$	20	125.0245(100)
3	Dihydroxybenzoic acid-pentoside	0.7	285.0619	1.1	$C_{12}H_{13}O_8$	30	108.0215(100); 152.0116(90)
4	Phenylalanine	1.0	164.0719	-1.2	$C_9H_{10}NO_2$	25	147.0442(100); 103.0550(33.7); 164.0719(12.6).
5	Pantotenic acid	1.1	218.1034	2.7	$C_9H_{16}NO_5$	10	218.1028(100); 216.0875(64,6); 146.0824(22.4)
6	Tryptophan	1.6	203.0827	-0.5	$C_{11}H_{11}N_2O_2$	20	116.0505(100); 142.0663 (32.1)
7	Methoxycinnamic acid	1.6	177.0557	-0.5	$C_{10}H_9O_3$	20	133.0653(100)
8	Chlorogenic acid	1.9	353.0878	-3.9	$C_{16}H_{17}O_9$	50	191.0565(100); 135.0450(9.2); 127.0405(8.6)
9	Caffeic acid	1.7	179.0346	2.1	$C_9H_7O_4$	20	135.0451(100)
10	(epi)Gallocatechin	2.4	305.0665	0.6	$C_{15}H_{13}O_7$	20	125.0243(100); 167.0349(44.1); 305.0665(43.1); 219.0662(34.2); 221.0452(17.6); 261.0768(17.1); 237.0769(6.8)
11	Coumaric acid	2.6	163.0401	0.2	$C_9H_7O_3$	30	119.0506(100)
12	Coumaric acid-hexoside	3.0	325.0917	3.7	$C_{15}H_{17}O_8$	10	163.0394(100); 119.0495(3.6)
13	Quinic acid-coumaroyl	3.2	337.0915	4.1	$C_{16}H_{17}O_8$	20	191.0566(100); 173.0458(29.4); 163.0404(13.0)
14	(epi)afzelechin-(epi)galocatechin	3.1	575.1182	2.3	$C_{30}H_{23}O_{12}$	20	303.0518(100); 285.0400(71.9); 439.0667(26.3); 125.0247(28.5); 245.0089(17.6)
15	(epi)afzelechin-(epi)catechin I	4.0	561.1421	-3.3	$C_{30}H_{25}O_{11}$	30	289.0719(100); 290.0754(15.1); 245.08194(9.8); 125.0246(5.7); 137.0246(4.8)
16	Catechin	4.3	289.0716	0.6	$C_{15}H_{13}O_6$	20	245.0818(94.3); 203.0709(53.3); 125.0242(39.5); 205.0504(38.6); 151.0398(29.2)
17	(epi)Catechin-(epi)Catechin	4.5	577.1357	-1.0	$C_{30}H_{25}O_{12}$	20	305.0668(100); 425.0880(60.2); 289.0718(41.4); 407.0774(29.8); 451.1035(23.6); 125.0244(13.6)
18	(epi)afzelechin-(epi)catechin II	5.3	561.1420	-3.1	$C_{30}H_{25}O_{11}$	20	125.0248(100); 273.0772(96,6); 287.0564(32.1); 289.0719(23.7); 409.0937(13.7); 435.1095(9.5)
19	Medioresinol	6.0	387.1668	1.9	$C_{18}H_{27}O_9$	20	387.1668(100); 207.1030(68.8); 163.1132(21.7); 113.0246(15.9)
20	(epi)afzelechin-(epi)afzelechin	6.2	545.1449	0.8	$C_{30}H_{25}O_{10}$	20	273.0770(100); 271.0612(17.8); 312.0639(17.6); 274.0809(16); 164.0120(14.2)
21	Proantociadin C1	6.3	865.1995	-1.1	$C_{45}H_{37}O_{18}$	45	289.0718(100); 287.0558(91.5); 125.0248(84.1); 407.0774(58.4); 161.0245(31.4)

Table S5. LC-HRMS data of the 55 inferred compounds identified based on the data of Brucker spectral libraries (continued)

Compound ID	Compound name	Rt (min)	*m/z* Experimental [M-H]$^-$	Error (ppm)	Molecular formula [M-H]$^-$	Collision Energy (eV)	Fragment ions (%)
22	afzelechin(4→8aAfzelechin(II)	6.6	545.1432	3.9	$C_{30}H_{25}O_{10}$	30	273.0771(100); 125.0243(17.4); 164.0115(16.6); 312.0643(14.0)
23	Orientin	6.7	447.0947	-5.6	$C_{21}H_{19}O_{11}$	30	327.0516(100); 357.0618(48.9); 297.0401(10.9); 285.0408(5.3); 229.0562(4.9); 339.0518(3.9)
24	Kaempferol-hexose-deoxyhexose	6.8	593.1502	-0.3	$C_{27}H_{29}O_{15}$	20	285.0406(100); 447.0919(48.1)
25	Myricetin-pentose(II)	6.9	449.0754	-6.3	$C_{20}H_{17}O_{12}$	40	316.0223(100); 271.0243(27.2); 317.0272(22.7); 287.0193(15.4); 178.9982(5.0); 151.0034(4.2)
26	Catechin gallate	7.0	441.0832	-1.1	$C_{22}H_{17}O_{10}$	40	169.0140(100); 125.0241(79.8); 245.0814(15.4); 289.0709(13.6); 203.0715(11.1); 137.0242(9.3); 151.0396(9.3)
27	Myricitrin	7.3	463.0874	-0.6	$C_{21}H_{19}O_{12}$	40	316.0221(100);271.0243(25.1); 287.0194(9.8); 151.0033(3.1)
28	Myricitin-215	7.4	531.0753	-5.9	$C_{31}H_{15}O_{9}$	20	316.0224(100); 271.0242(15.9); 287.0199(7.9); 178.9984(4.3); 151.0034(3.5); 137.0238(1.4)
29	Quercetin-441	7.5	741.1896	1.2	$C_{32}H_{37}O_{20}$	50	300.0277(100); 178.9983(2.8); 271.0248 (2.2); 255.0295(1.1); 151.0034(1.0)
30	Isovitexin	7.6	431.0998	-3.3	$C_{21}H_{19}O_{10}$	30	311.0574(100); 283.0618(14.1); 341.0683(9.6); 323.0563(2.4)
31	Peltatoside	7.7	595.1316	-1.9	$C_{26}H_{27}O_{16}$	20	300.0276(100); 301.0322(22.0); 178.9987(1.6); 255.0296(1.1); 151.0035(0.7); 463.0890(0.5)
32	Kaempferol-deoxyhexose-deoxyhexose-hexose	7.8	739.2126	-4.7	$C_{33}H_{39}O_{19}$	50	284.0331(100); 255.0303(4.1); 178.9986(1.5); 227.0350(1.7); 151.0038(1.2)
33	Quercetin-309	7.8	609.1461	-1.3	$C_{27}H_{29}O_{16}$	40	300.0276(100); 271.0248(2.3); 178.9985(1.6); 255.0296(0.9); 151.0031(0.8)
34	Quercetin-hexose	7.8	463.0886	-0.9	$C_{21}H_{19}O_{12}$	30	300.0277(100); 271.0249(2.9);178.9985(1.6); 151.0038(1.4)
35	Isorhamnetin-471	7.9	785.2153	-0.9	$C_{34}H_{31}O_{21}$	50	314.0430(100); 315.0496(54.3); 299.0198(9.3); 316.0535(8.6); 300.0247(4.8); 178.9979(2.9)
36	Kaempferol-308	8.1	593.1536	-4.1	$C_{27}H_{29}O_{15}$	50	284.0331(100); 285.0388(31.8); 227.0351(10.2); 151.0040(1.4)
37	NCGC00384841	8.1	539.2110	4.5	$C_{26}H_{35}O_{12}$	30	491.1924(100); 165.0556(32.1); 195.0656(21); 343.1390(20.6); 329.1393(19.7)
38	Azelaic acid	8.2	187.0969	3.6	$C_{9}H_{15}O_{4}$	20	125.0964(100); 169.0860(10.8)
39	Vitexin	8.3	431.0985	-0.3	$C_{21}H_{19}O_{10}$	40	311.0563(100); 283.0612(99.9); 164.0112(31.7); 341.0668(31); 323.0561(20.8)
40	Methylquercetin-455	8.4	769.2211	-1.9	$C_{34}H_{41}O_{20}$	50	314.0433(100); 299.0198(0.8); 178.9982(2.3); 151.0034(1.2)
41	Avicularin (quercetin-3-O-arabinofuranoside)	8.5	433.0774	0.5	$C_{20}H_{17}O_{11}$	40	300.0246(100); 271.0220(45.2); 255.0270(19.3); 151.0017(7.1)
42	Kaempferol-308(II)	8.5	593.1537	-4.2	$C_{27}H_{29}O_{15}$	45	284.0330(100); 255.0300(10.3); 227.0353(3.5)

Table 5S. LC-HRMS data of the 55 inferred compounds identified based on the data of Brucker spectral libraries (continued)

Compound ID	Compound name	Rt (min)	*m/z* Experimental $[M-H]^-$	Error (ppm)	Molecular formula $[M-H]^-$	Collision Energy (eV)	Fragment ions (%)
43	Quercetrin (Quercetin 3-O-rhamnoside)	9.1	447.0907	5.8	$C_{21}H_{19}O_{11}$	40	300.0250(100); 284.0302(21.6); 151.0026(2.6); 178.9971(2.7)
44	(epi)afzelechin-(epi)catechin(II)	9.2	561.1430	-4.9	$C_{30}H_{25}O_{11}$	30	289.0720(100); 271.0615(11.9); 245.0823(10.1); 137.0246(7.2); 125.0243(6.3)
45	Isorhamnetin-hexose	9.9	477.1038	1.2	$C_{22}H_{21}O_{12}$	40	314.0429(100); 243.0294(34.9); 271.0243(32.8); 285.0401(29.9); 257.0451(13.8)
46	Naringenin 7-O-glucoside	10.1	433.1160	-4.6	$C_{21}H_{21}O_{10}$	35	271.0622 (100); 268.0391(66.7); 151.0046(37.6); 119.0514(10.1)
47	Kaempferol-131	10.3	415.1946	6.6	$C_{20}H_{31}O_{9}$	30	284.0321(100); 137.0243(29.8); 151.0410(22.9); 125.0246(17.4); 227.0346(15.3); 255.0297(14.4)
48	3',4',7,8-Tetrahydroxyflavanone	10.4	287.0561	0.0	$C_{15}H1_{1}O_{6}$	20	151.0039(100); 135.0454(60.9); 283.2640(8.4)
49	Kaempferol-214	10.5	499.0860	4.4	$C_{24}H_{19}O_{12}$	40	285.0398(100); 255.0295(4.2); 227.0350(1.6)
50	Kaempferide-116	10.7	417.2119	-1.7	$C_{20}H_{33}O_{9}$	40	284.0317(100); 301.0367(91.5); 255.0284(81.0); 227.0355(60.9); 151.0396(54.4)
51	Quercetin-313	10.8	475.0874	1.7	C22H19O12	20	300.0276(100); 285.0402(35.8); 271.0245 (3.0); 255.0295(1.8); 178.9986(1.1)
52	Trihydroxyflavone-dimethyl-161	11.7	461.1450	0.7	$C_{23}H_{25}O_{10}$	20	269.0455(100); 284.0685(75); 241.0506(64.9); 225.0552(25.7); 240.0419(22.3)
53	7,4'- Dimethoxy-5-hydroxyflavone-203	11.7	503.1558	0.6	$C_{25}H_{27}O_{11}$	40	284.0690(100);269.0459(34.2); 299.0925(27.2); 241.0506(19.6)
54	Naringenin Falcone	12.1	271.0614	-0.7	$C_{15}H_{11}O_{5}$	30	119.0502(100); 151.0035(60.5); 107.0132(16.0); 187.0396
55	Bauhiniastatin 2	14.6	299.0917	-5.3	$C_{17}H_{15}O_{5}$	30	225.0556(100); 197.0605(83.1); 241.0505(75.3); 210.0320(36.1)

Table S6. Presence of the compounds in the ethanolic extracts of leaves of *B. forficata*, *B. variegata*, *B. longifolia*, and *B. affinis*, at the experimental conditions evaluated for sample preparation and analysis

Compound ID	Compound	*B. forficata*	*B. longifolia*	*B. variegata*	*B. affinis*
1	Hexose-hexose	X	X	X	X
2	Gallic acid		X	X	
3	Dihydroxybenzoic acid-pentoside	X	X	X	X
4	Phenylalanine	X	X	X	X
5	Pantotenic acid	X	X	X	X
6	Tryptophan	X	X	X	X
7	Methoxycinnamic acid				X
8	Chlorogenic acid			X	
9	Caffeic acid	X	X	X	
10	(epi)Gallocatechin		X	X	
11	Coumaric acid	X	X	X	X
12	Coumaric acid-hexoside	X	X	X	X
13	Quinic acid-coumaroyl	X		X	
14	(epi)afzelechin-(epi)galocatechin		X	X	
15	(epi)afzelechin-(epi)catechin I	X		X	
16	Catechin	X	X	X	
17	(epi)catechin-(epi)catechin	X	X	X	
18	(epi)afzelechin-(epi)catechin II	X	X	X	
19	Medioresinol	X	X	X	X
20	(epi)afzelechin-(epi)afzelechin	X		X	
21	ProantociadinC1		X	X	
22	afzelechin(4→8)afzelechin(II)	X	X	X	
23	Orientin	X		X	

Table S6. Presence of the compounds in the ethanolic extracts of leaves of *B. forficata*, *B. variegata*, *B. longifolia*, and *B. affinis*, at the experimental conditions evaluated for sample preparation and analysis (continued)

Compound ID	Compound	*B. forficata*	*B. longifolia*	*B. variegata*	*B. affinis*
24	Kaempferol-hexose-deoxyhexose		X		
25	Myricetin-pentose(II)		X		
26	Catechin gallate		X		
27	Myricitin		X		
28	Myricitrin-215		X		
29	Quercetin-441		X		
30	Isovitexin	X		X	
31	Peltatoside	X		X	
32	Kaempferol-deoxyhexose-deoxyhexose-hexose	X		X	
33	Quercetin-309	X	X	X	X
34	Quercetin-hexose	X	X	X	X
35	Isorhamnetin-471	X		X	
36	Kaempferol-308		X		
37	NCGC00384841		X		
38	Azelaic acid	X	X	X	X
39	Vitexin		X		
40	Methylquercetin-455	X	X		
41	Avicularin (quercetin-3-O-arabinofuranoside)	X	X	X	X
42	Kaempferol-308(II)	X	X	X	X
43	Quercetrin (Quercetin 3-O-rhamnoside)	X	X	X	X
44	(epi)afzelechin-(epi)catechin(II)	X	X	X	
45	Isorhamnetin-hexose	X		X	
46	Naringenin 7-O-glucoside			X	
47	Kaempferol-131	X	X	X	
48	3',4',7,8-Tetrahydroxyflavanone		X		

Table S6. Presence of the compounds in the ethanolic extracts of leaves of *B. forficata*, *B. variegata*, *B. longifolia*, and *B. affinis*, at the experimental conditions evaluated for sample preparation and analysis (continued)

Compound ID	Compound	*B. forficata*	*B. longifolia*	*B. variegata*	*B. affinis*
49	Kaempferol-214		X		
50	Kaempferide-116	X	X	X	X
51	Quercetin-313		X		
52	Trihydroxyflavone-dimethyl-161	X	X	X	
53	7,4’- Dimethoxy-5-hydroxyflavone -203		X		X
54	Naringenin Falcone		X		
55	Bauhiniastatin 2	X	X	X	X

SECTION 1. Secondary Metabolites Chemical Characterization by LC-HRMS

1.1. Hexose-Hexose

The hexose-hexose (**1**) disaccharide (other isomers) was identified according to fragmentation of the deprotonated molecular ion $[M-H]^-$ at *m/z* 341.1077 ($C_{12}H_{21}O_{11}$), which produced fragment ions at *m/z* 179.0564 and *m/z* 161.0453, attributed to the monosaccharide. Also, the spectrum of this disaccharide was compared with literature data (Matsuda et al., 2010; Matsusa et al., 2016; Valgimigli et al., 2012).

1.2. Organic acids and aminoacids

Compound **(3)** was characterized as dihydroxybenzoic acid-pentoside with a deprotonated molecular ion at *m/z* 285.0619, yielding fragment ions at *m/z* 108.0214 (loss of HCO_2) and *m/z* 109.0281 $[M-H-44]^-$ (loss of CO_2), depending on the collision energy dissociation applied, which is a characteristic of the dihydroxybenzoic acid and the isomers gentisic acid and protocatechuic acids, both previously reported for the genus Bauhinia (Compaoré et al., 2012; Nageshwar et al., 1986). Additionally, the compound **(3)** also produced fragment ions at *m/z* 153.0192, 152.0117 and loss of pentose $[M-H-132]^-$, being another characteristic of dihydroxybenzoic acid-pentoside. Pantothenic acid (**5**) was inferred based on the exact mass at *m/z* 218.1026 and by comparison of the fragment ion at *m/z* 146.0816 (loss of $C_3H_4O_2$) with the MassBank database. (Kakazu & Horai, 2016)

Phenylalanine **(4)** was identified based on the exact mass at *m/z* 164.0709 and fragment ions at *m/z* 147, which is related to the loss of one amino group and one hydrogen atom from the benzylic ring. This phenomenon may occur due to the rearrangement of a proton from the benzylic position to a carboxyl group through a five-membered ring transition state, resulting in the formation of an intermediate having a carbanion at the benzyl position. By increasing the collision dissociation energy, the relative intensity of the fragment ion at *m/z* 103 increases, while the fragment ion at *m/z* 147 decreases. This result suggests that the fragment ion at *m/z* 147 has a high internal energy and can be further fragmented via the loss of CO_2 (44 Da) to form the fragment ion at *m/z* 103. (Matsuda et al., n.d.; Sekimoto et al., 2014)

Tryptophan's (**6**) identification was based on the fragment ions at *m/z* 116, which represents the indole ion of the molecule obtained by loss of the sidechain (Kakazu & Horal, 2016; Lambert et al.,

2015). The Methoxycinamic acid (**7**) were identified based on the loss of CO_2 (44 Da) from *m/z* 177.0558, yielding the fragment ion *m/z* 133. (Metlin 6453, n.d.)

Chlorogenic acid (**8**) and quinic acid-cumaroyl (**13**), with the molecular formulas $C_{16}H_{18}O_9$ $[M-H]^-$ *m/z* 353.0892 and $C_{16}H_{18}O_8$ $[M-H]^-$ *m/z* 337.0915, respectively, were identified due to fragmentation by charge retention fragmentation (CRF) via remote hydrogen rearrangements (RHR), which promoted the neutral loss of the corresponding part of the molecule to caffeic acid (9) and coumaric acid (**11**), respectively. Also, the chlorogenic acid showed the fragment ion at *m/z* 191.0568 that is relative to quinic acid and the quinic-coumaroil acid showed fragment ions at m/z 163.0403 and 119.0503 due to the fragmentation of the coumaric acid {Formatting Citation}.

1.3. Flavonoid O-glycosides, C-glycosides, and other compounds (25, 27, 29-30, 32-36, 40-43, and 45-54)

The fragment ions at *m/z* 284/285, 300, 314, 316 were characterized as the radical aglycon $[Y_0-H]^{\bullet -}$ and/or the aglycon ions $[Y_0]^{--}$ in the negative ionization mode (Aquino et al., 2019), from the flavonoid aglycones kaempferol (compounds **24, 32, 36, 42, 47**, and **49**), quercetin (**29, 33, 34, 43**, and **51**), methylquercetin (**40**), and myricetin (**25, 27**, and **28**), respectively. The complete fragmentation studies and the flavonoid's spectra have been previously published (Aquino et al., 2019; Aquino & http://lattes.cnpq.br/6601636992092317, 2018).

Additionally, kaempferitrin, an alleged chemical marker of Bauhinia forficate, which shows a deprotonated molecular ion at *m/z* 577.1552 and adduct ions ($[M-AF]^-$ and $[M-H_2O-H]$, and clusters ($[2M-H]^-$, $[3M-H]^-$, $[2M+AF-H]^-$) was not found in any of these forms. These data corroborate those previously published by Ferreres *et al.* (Ferreres et al., 2012), who also studied B. forficata Link subspecies pruinosa (Vogel) Fortunato & Wunderlin and did not found this substance.

Bauhiniastatin 2 is present in all species studied in this work, as illustrated in Table (7). This compound has been reported in *B. purpurea*, and its medicinal properties are related to anticancer activity, which demonstrates the potential of these 4 species of *Bauhinia* for this purpose.

1.4. Proanthocyanidin (Type B and A) and Procyanidin C1(14-18, 20, 22, and 44)

The identification of the proanthocyanidin type B dimers was based on their fragmentation patterns (Li and Deinzer, 2007; Demarque et al., 2016). For example, (epi)catechin-(epi)catechin (**17**),

(proanthocyanidin type B) exhibited a deprotonated molecular ion at *m/z* 577.1365 and a fragment ion at *m/z* 425, from a retro-Diels-Alder (RDA) cleavage. The fragment ions *m/z* 451.1035 and 289.0718 were obtained via ε-elimination indicating carbon-carbon bonding unit and (epi) catechin, respectively. The peak (**14**) was attributed to (epi) afzelechin-(2→7,4→8) (epi) gallocatechin, protoantocyanidin type A, due to the presence of fragment ions at *m/z* 303.0508 and 439.0663 and exact mass *m/z* 575.1115 (Li and Deinzer, 2007).

The fragmentation of the trimer (proanthocyanidin C1, compound **21** in table 6) was to be like the dimers. The fragment ion ($[M-H-152]^-$) at *m/z* 713.1 was derived from an RDA reaction of B-type procyanidin trimer, and the fragment ion ($[M-H-288]^-$) at *m/z* 577.1 was originated from the cleavage of the B-type trimer, which could take place at either the upper interflavonoid bond or the lower bond (Karonen et al., 2004; Li et al., 2012). Besides, fragment ions were detected at *m/z* 425.0, 407.1, and 288.9. The fragment ions ($[M-H-440]^-$) at *m/z* 425.0 was originated from an RDA, while the $[M-H-458]^-$ with a fragment ion at *m/z* 407.1, owing to a loss of water.

1.5. Others

The compound **38** (azelaic acid/nonanedioic acid – medium-chain fatty acid) showed the following deprotonated molecular ion $[M-H]^-$ at *m/z* 187.098 and fragment ion at *m/z* 125.0975 (Metlin). The fragment ions of NCGC00384841 (**37**) were *m/z* 491.1924 and 343.1390. For Medioresinol (**19**), the fragment ions were at *m/z* 163 and 207. (Mona; Bonzanini et al., 2009; Bendif et al., 2020)

Section 2. Metabolite Spectra: Complete mass spectra of each compound are herein organized by retention time (Rt min), measured *m/z*, ion formula, error (ppm), and collision dissociation energy.

Compound 1 - Hexose-hexose

UHPLC (Rt min)	Measured *m/z*	Ion Formula $[M-H]^-$	Theoretical *m/z*	Error (ppm)	eV(MS^-)
0.5	341.1093	$C_{12}H_{21}O_{11}$	341.1089	1.2	20

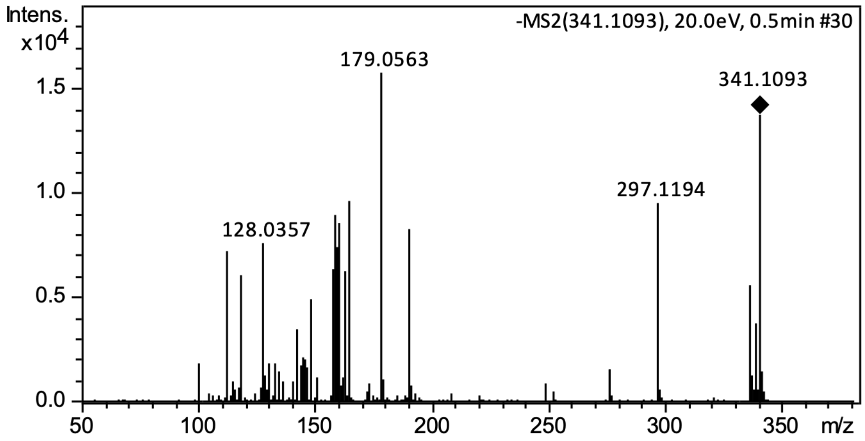

m/z	I %
179.0563	100.0
341.1093	87.3
165.0403	61.1
297.1194	60.2
159.0301	56.4
161.0461	54.1
191.0563	52.7
128.0357	48.3
160.0617	47.0
113.0249	45.4
158.0459	40.0
119.0350	38.1
143.0352	22.1

Compound 2 - Galic-Acid

UHPLC (Rt min)	Measured *m/z*	Ion Formula $[M-H]^-$	Theoretical *m/z*	Error (ppm)	eV(MS$^-$)
0.60	169.0140	$C_7H_5O_5$	169.0142	-1.2	20

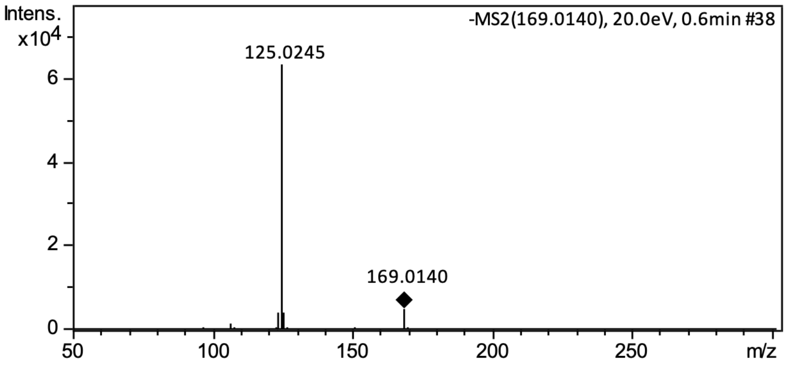

m/z	I %
125.0245	100.0
169.0140	7.4
124.0168	6.2
126.0279	6.1
107.0133	2.1
151.0038	1.0
127.0301	0.8

Compound 3 - Dihydroxybenzoic acid-pentoside

UHPLC (Rt min)	Measured *m/z*	Ion Formula $[M-H]^-$	Theoretical *m/z*	Error (ppm)	eV(MS$^-$)
0.68	285.0619	$C_{12}H_{13}O_8$	285.0616	1.0	20

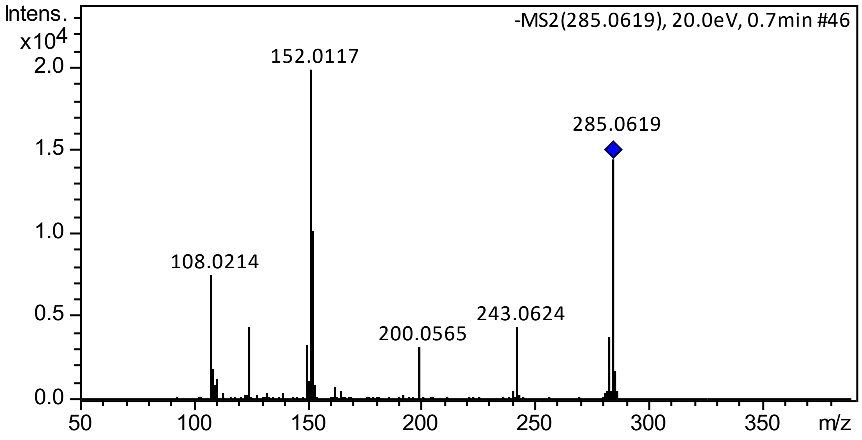

m/z	I %
152.0117	100.0
285.0619	72.6
153.0192	51.2
108.0214	37.8
243.0624	21.7
125.0246	21.6
283.2643	18.5
150.0421	16.4
200.0565	16.0
109.0281	8.8
286.0652	8.6
111.0197	6.2

Compound 4 - Phenylalanine

UHPLC (Rt min)	Measured *m/z*	Ion Formula $[M-H]^-$	*m/z*	Error (ppm)	eV(MS^-)
1.0	164.0712	$C_9H_{10}NO_2$	164.0717	-3.0	25

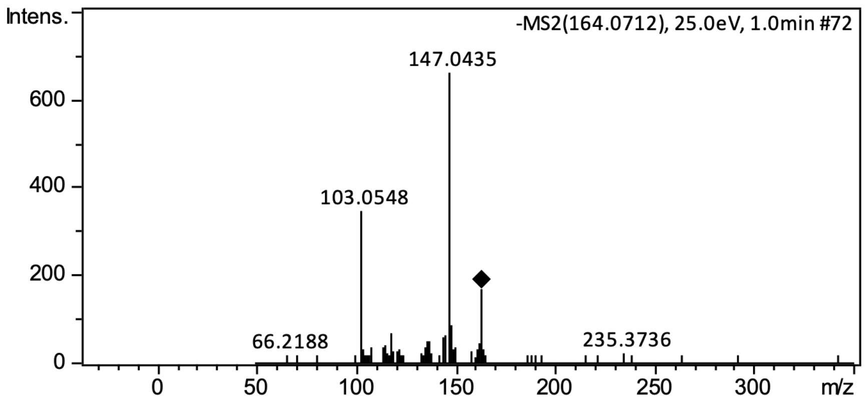

m/z	I %
147.0435	100.0
103.0548	52.3
164.0712	25.5

Compound 5 - Pantotenic acid

UHPLC (Rt min)	Measured *m/z*	Ion Formula $[M-H]^-$	*m/z*	Error (ppm)	eV(MS^-)
1.1	218.1028	$C_9H_{16}NO_5$	218.1034	-2.8	10

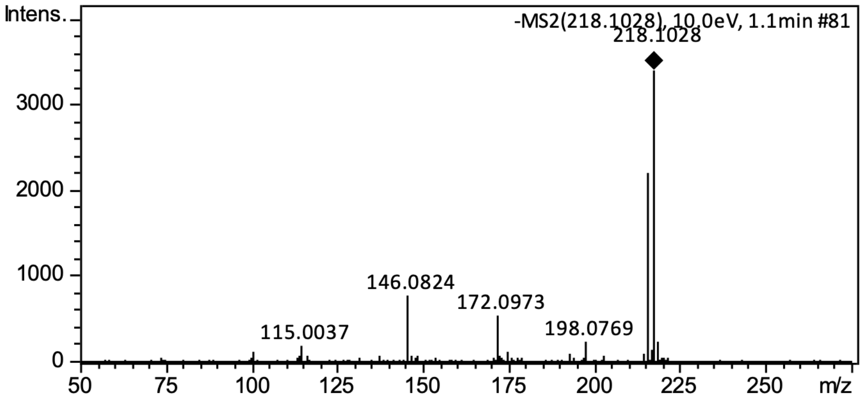

m/z	I %
218.1028	100.0
216.0875	64.6
146.0824	22.4
172.0973	16.1

Compound 6 - Tryptophan

UHPLC (Rt min)	Measured *m/z*	Ion Formula [M-H]$^-$	*m/z*	Error (ppm)	eV(MS$^-$)
1.6	203.0827	$C_{11}H_{11}N_2O_2$	203.0826	-0.5	20

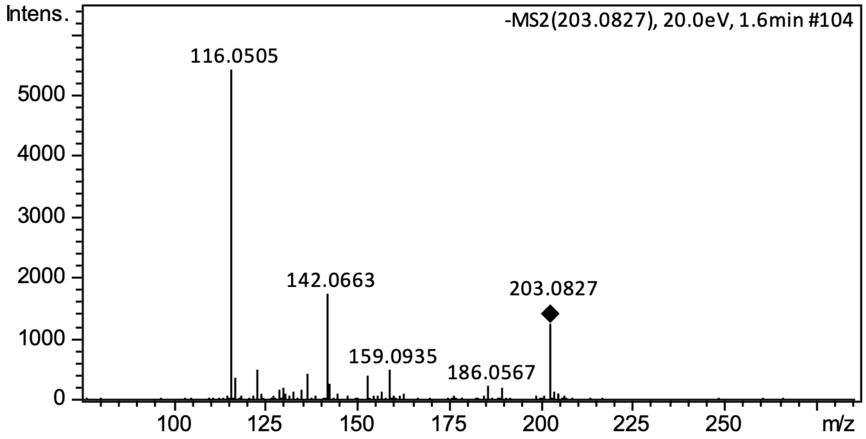

m/z	I %
116.0505	100.0
142.0663	32.1
203.0827	23.3
159.0935	9.3

Compound 7- Methoxycinnamic acid

UHPLC (Rt min)	Measured *m/z*	Ion Formula [M-H]$^-$	*m/z*	Error (ppm)	eV(MS$^-$)
1.6	177.0558	$C_{10}H_9O_3$	177.0557	-0.6	20

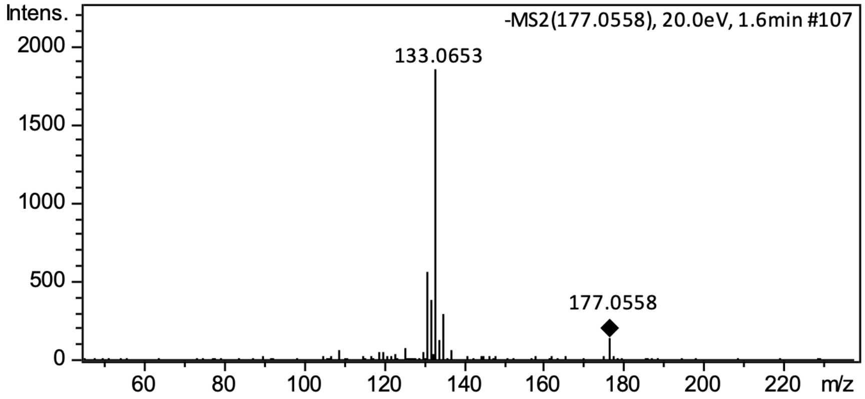

m/z	I %
133.0653	100.0
131.0496	30.6
132.0574	20.7
135.0442	15.8
177.0558	7.9

Compound 8 - Chlorogenic acid;

UHPLC (Rt min)	Measured *m/z*	Ion Formula $[M-H]^-$	*m/z*	Error (ppm)	$eV(MS^-)$
1.9	353.0892	$C_{16}H_{17}O_9$	353.0878	4.0	50

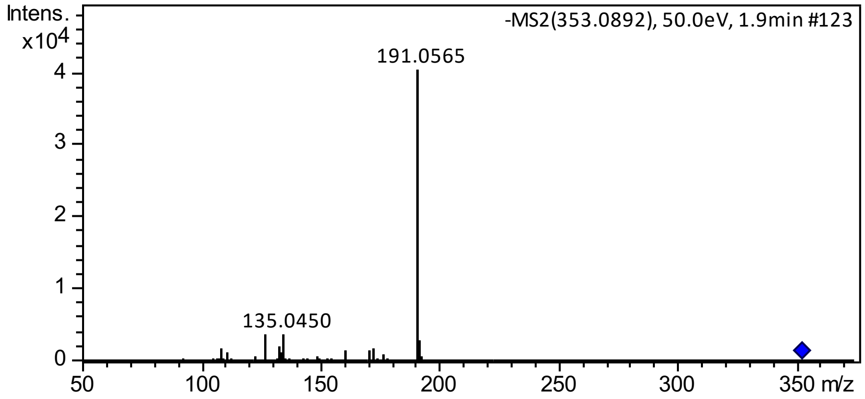

m/z	I %
191.0565	100.0
135.0450	9.2
127.0405	8.6
192.0605	6.8
133.0296	5.1
173.0461	4.2
109.0296	3.9
161.0251	3.8

Compound 9 - Caffeic acid

UHPLC (Rt min)	Measured *m/z*	Ion Formula $[M-H]^-$	*m/z*	Error (ppm)	$eV(MS^-)$
1.7	179.0346	$C_9H_7O_4$	179.0350	-2.2	20

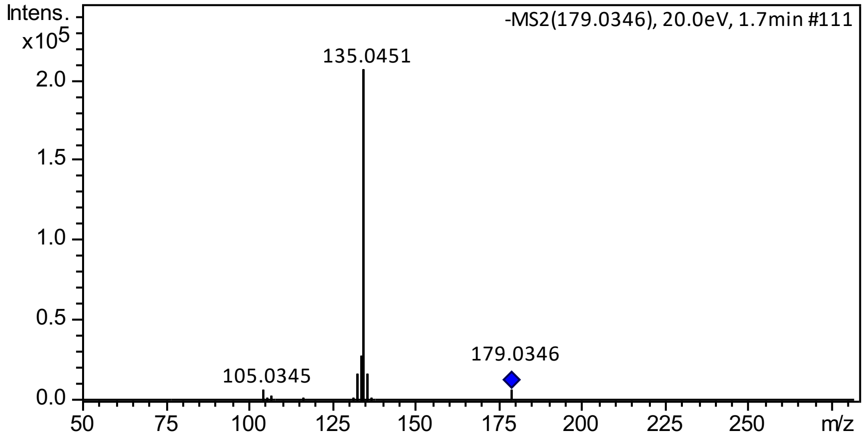

m/z	I %
135.0451	100.0
134.0371	13.2
133.0295	7.7
136.0484	7.6
179.0346	3.1

Compound 10 - (epi)Gallocatechin

UHPLC (Rt min)	Measured *m/z*	Ion Formula [M-H]$^-$	*m/z*	Error (ppm)	eV(MS$^-$)
2.40	305.0665	$C_{15}H_{13}O_7$	305.0667	-0.7	20

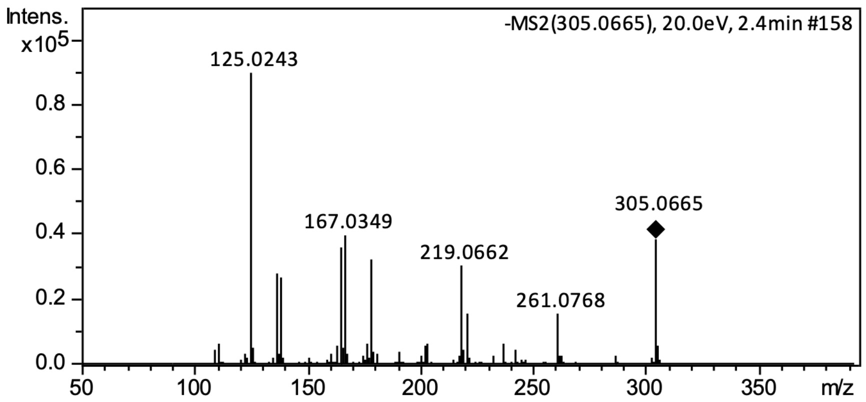

m/z	I %
125.0243	100.0
167.0349	44.1
305.0665	43.1
165.0190	40.2
179.0348	35.9
219.0662	34.2
137.0243	31.4
139.0398	29.8
221.0452	17.6
261.0768	17.1
111.0449	7.2
204.0422	7.0
237.0769	6.8

Compound 11 - Coumaric acid

UHPLC (Rt min)	Measured *m/z*	Ion Formula [M-H]$^-$	*m/z*	Error (ppm)	eV(MS-)
2.6	163.0401	$C_9H_7O_3$	163.0401	0.0	30

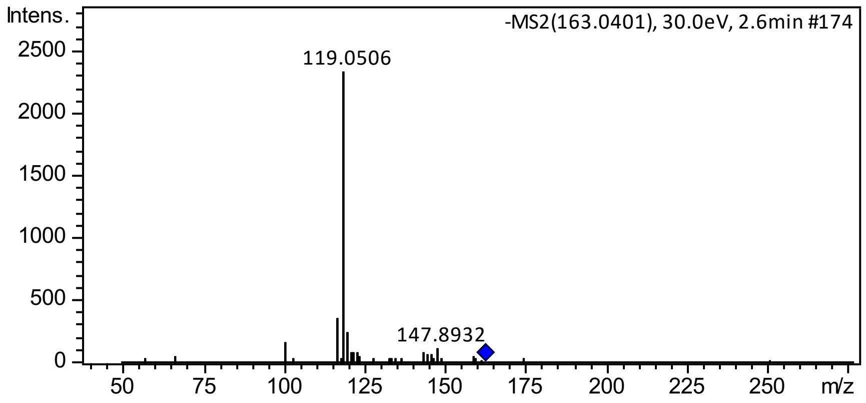

m/z	I %
119.0506	100.0
117.0348	15.3
120.0526	10.3
101.0380	6.7
147.8932	5.0

Compound 12 - Coumaric acid-hexoside

UHPLC (Rt min)	Measured *m/z*	Ion Formula $[M-H]^-$	*m/z*	Error (ppm)	eV(MS-)
3.0	325.0917	$C_{15}H_{17}O_8$	325.0928	-3.4	10

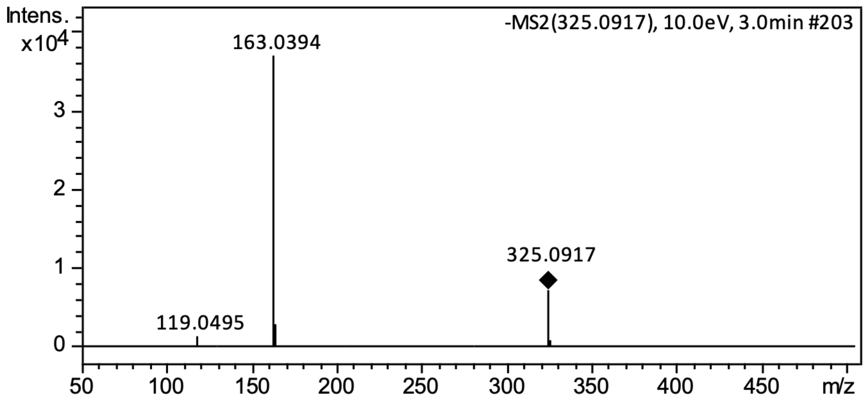

m/z	I %
163.0394	100.0
325.0917	19.6
164.0428	7.9
119.0495	3.6
326.0954	2.4

Compound 13 - Quinic acid-coumaroyl

UHPLC (Rt min)	Measured *m/z*	Ion Formula $[M-H]^-$	*m/z*	Error (ppm)	$eV(MS^-)$
3.00	337.0915	$C_{16}H_{17}O_8$	337.0929	-4.2	20

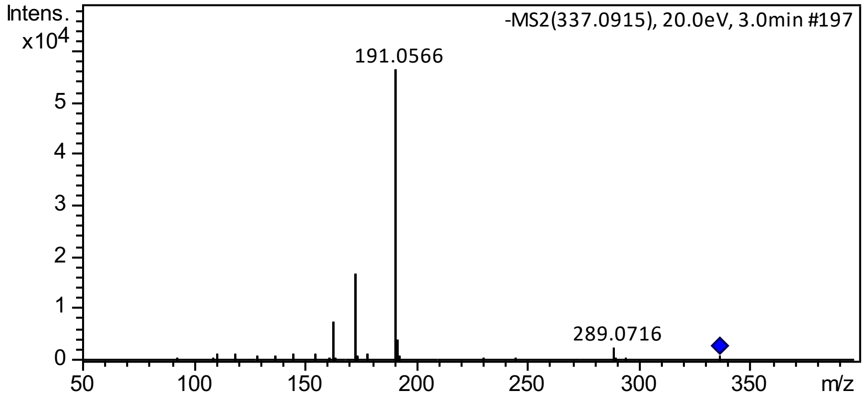

m/z	I %
191.0566	100.0
173.0458	29.4
163.0404	13.0
192.0594	6.7
289.0716	4.0
119.0497	2.3
111.0446	2.2
179.0351	2.0
155.0343	1.8
145.0297	1.7
337.0915	1.5
174.0499	1.5

Compound 14 - (epi)afzelechin-(epi)galocatechin

UHPLC (Rt min)	Measured *m/z*	Ion Formula [M-H]$^-$	*m/z*	Error (ppm)	eV(MS$^-$)
3.11	575.1182	$C_{30}H_{23}O_{12}$	575.1195	-2.3	20

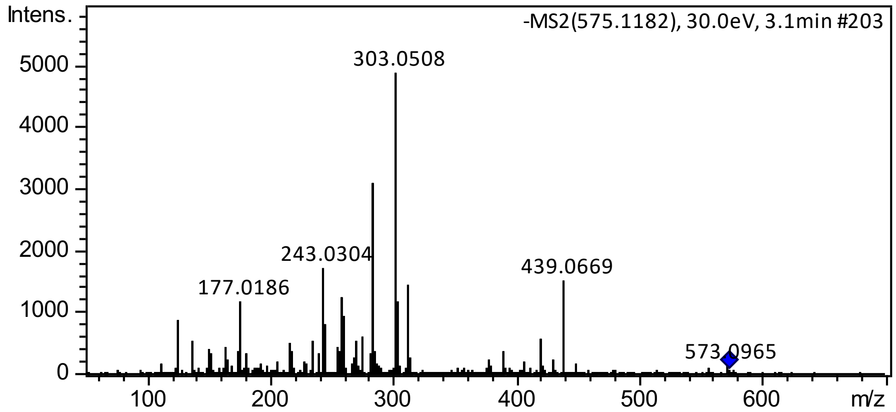

m/z	I %
303.0518	100.0
285.0400	71.9
243.0301	36.6
125.0247	28.5
177.0197	27.1
439.0667	26.3
313.0357	26.2
259.0610	22.7
245.0089	17.6
261.0398	17.6
304.0545	14.9
286.0448	14.1
275.0553	14.0

Compound 15 - (epi) afzelechin-(epi)catechin I

UHPLC (Rt min)	Measured *m/z*	Ion Formula [M-H]$^-$	*m/z*	Error (ppm)	eV(MS-)
4.00	561.1421	$C_{30}H_{25}O_{11}$	561.1402	3.4	30

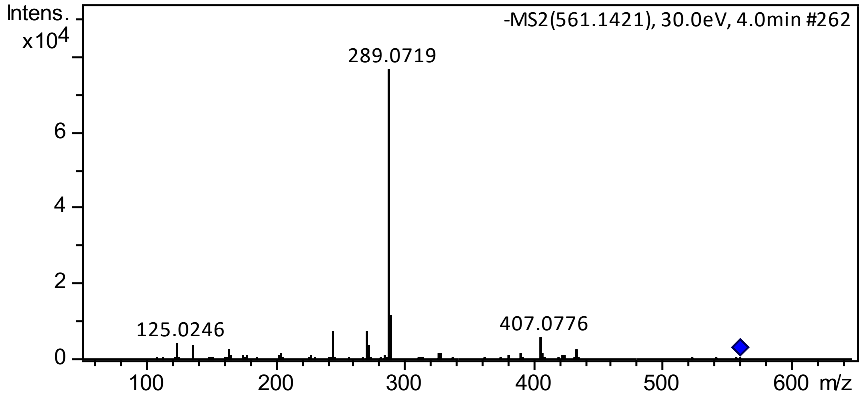

m/z	I %
289.0719	100.0
290.0754	15.1
245.0819	9.8
271.0618	9.3
407.0776	7.2
125.0246	5.7
273.0766	4.9
137.0246	4.8
435.1081	3.7
164.0118	3.6
165.0183	2.6

Compound 16 - Catechin

UHPLC (Rt min)	Measured *m/z*	Ion Formula $[M-H]^-$	*m/z*	Error (ppm)	eV(MS-)
4.27	289.0716	$C_{15}H_{13}O_6$	289.0718	-0.7	20

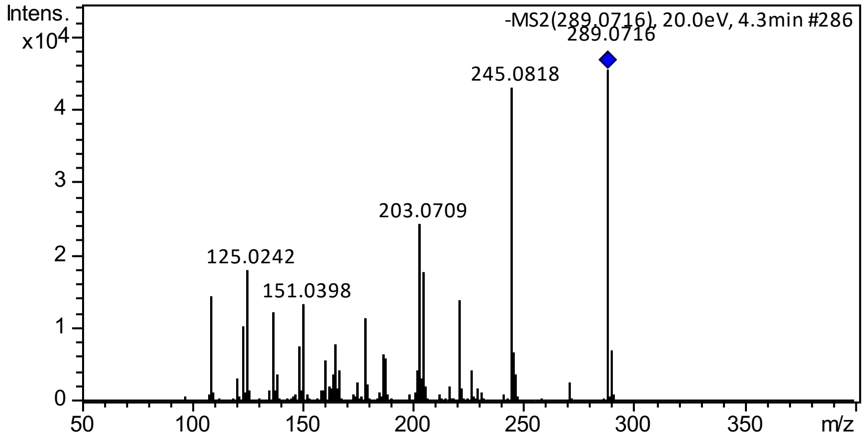

m/z	I %
289.0716	100.0
245.0818	94.3
203.0709	53.3
125.0242	39.5
205.0504	38.6
109.0291	31.3
221.0817	30.0
151.0398	29.2
137.0243	26.7
179.0348	24.9
123.0450	22.4

Compound 17 - (epi)Catechin-(epi)Catechin

UHPLC (Rt min)	Measured *m/z*	Ion Formula $[M-H]^-$	*m/z*	Error (ppm)	$eV(MS^-)$
4.50	577.1357	$C_{30}H_{25}O_{12}$	577.1351	1.0	20

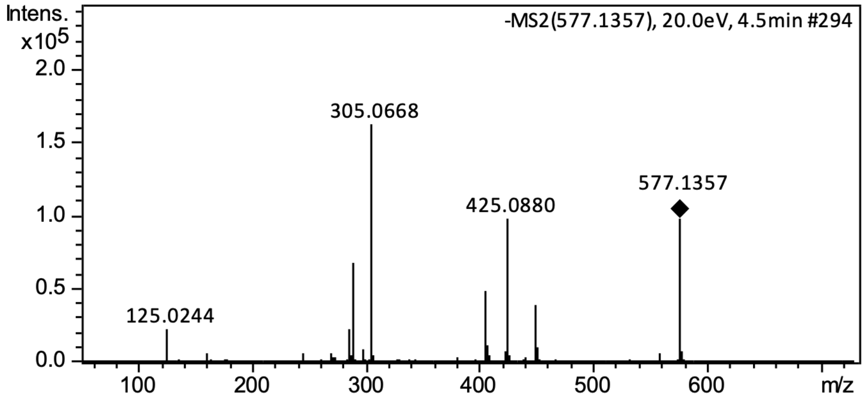

m/z	I %
305.0668	100.0
425.0880	60.2
577.1357	60.0
289.0718	41.4
407.0774	29.8
451.1035	23.6
578.1389	19.3
306.0703	16.1
287.0560	13.8
125.0244	13.6
426.0912	13.1
290.0752	6.6
408.0809	6.5

Compound 18 - (epi) afzelechin-(epi)catechin II

UHPLC (Rt min)	Measured *m/z*	Ion Formula [M-H]$^-$	*m/z*	Error (ppm)	eV(MS$^-$)
5.26	561.1420	$C_{30}H_{25}O_{11}$	561.1402	3.2	20

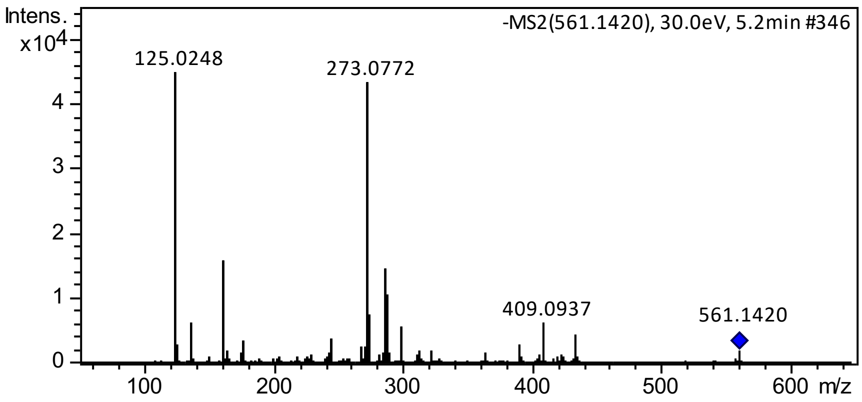

m/z	I %
125.0248	100.0
273.0772	96.6
161.0248	35.2
287.0564	32.1
289.0719	23.7
274.0808	16.2
137.0249	13.7
409.0937	13.6
299.0563	12.2
435.1095	9.5
245.0456	8.1
177.0199	7.6
126.0282	6.5

Compound 19 - Medioresinol

UHPLC (Rt min)	Measured *m/z*	Ion Formula [M-H]$^-$	*m/z*	Error (ppm)	eV(MS$^-$)
6.02	387.1668	$C_{18}H_{27}O_9$	387.1661	1.8	20

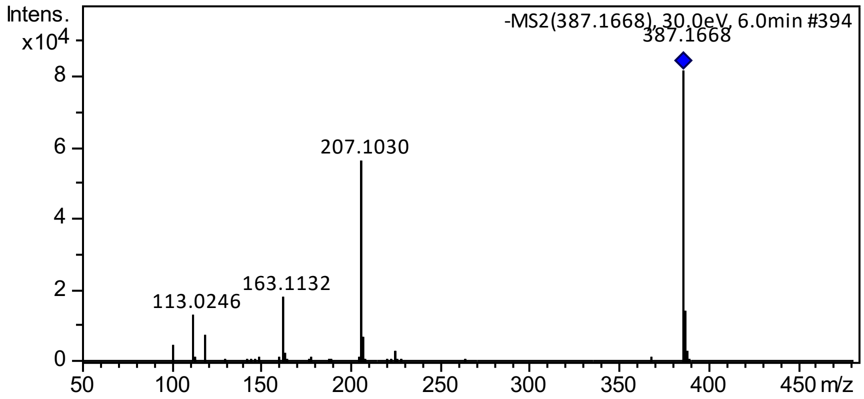

m/z	I %
387.1668	100.0
207.1030	68.8
163.1132	21.7
388.1702	16.9
113.0246	15.9
119.0352	8.6
208.1064	8.3
101.0245	5.2
389.1727	3.5
225.1141	3.5

Compound 20 - (epi)afzelechin-(epi)afzelechin

UHPLC (Rt min)	Measured *m/z*	Ion Formula $[M-H]^-$	*m/z*	Error (ppm)	$eV(MS^-)$
6.21	545.1449	$C_{30}H_{25}O_{10}$	545.1453	-0.7	20

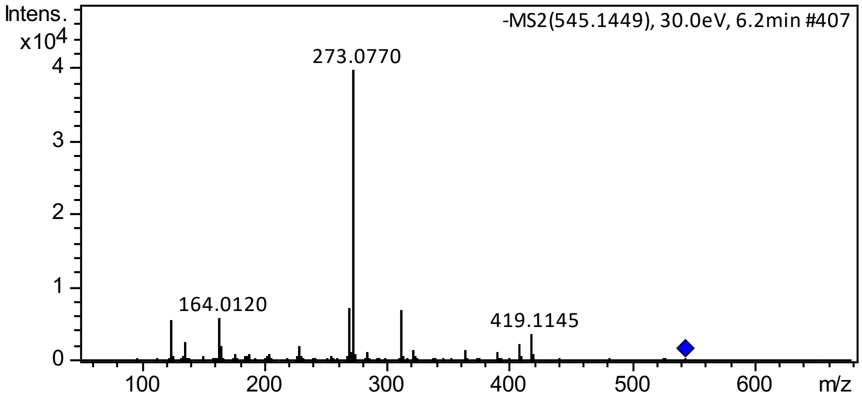

m/z	I %
273.0770	100.0
271.0612	17.8
312.0639	17.6
274.0809	16.0
164.0120	14.2
125.0243	13.6
419.1145	9.1
313.0703	9.0
137.0247	6.0

Compound 21 - Proantociadina C1

UHPLC (Rt min)	Measured *m/z*	Ion Formula $[M-H]^-$	*m/z*	Error (ppm)	$eV(MS^-)$
6.25	865.1995	$C_{45}H_{37}O_{18}$	865.1985	1.2	45

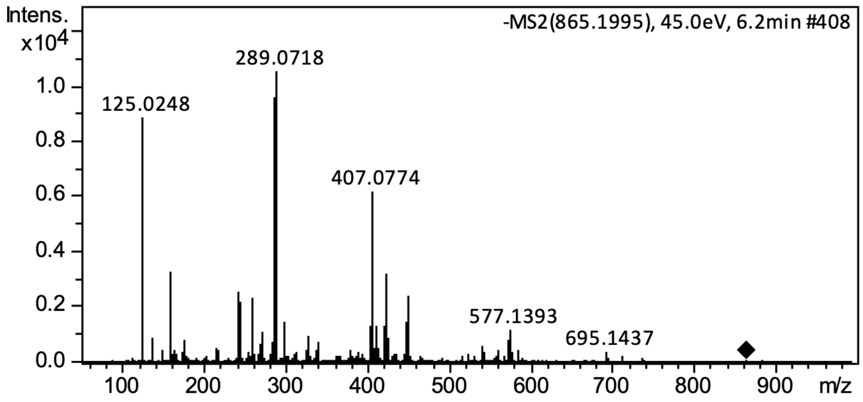

m/z	I %
289.0718	100.0
287.0558	91.5
125.0248	84.1
407.0774	58.4
161.0245	31.4
425.0891	30.0
243.0297	24.4
261.0406	22.4
451.1038	22.4
245.0463	20.6

Compound 22 - afzelechin(4→8)afzelechin(II)

UHPLC (Rt min)	Measured *m/z*	Ion Formula [M-H]⁻	*m/z*	Error (ppm)	eV(MS⁻)
6.83	545.1432	$C_{30}H_{25}O_{10}$	545.1453	-3.9	30

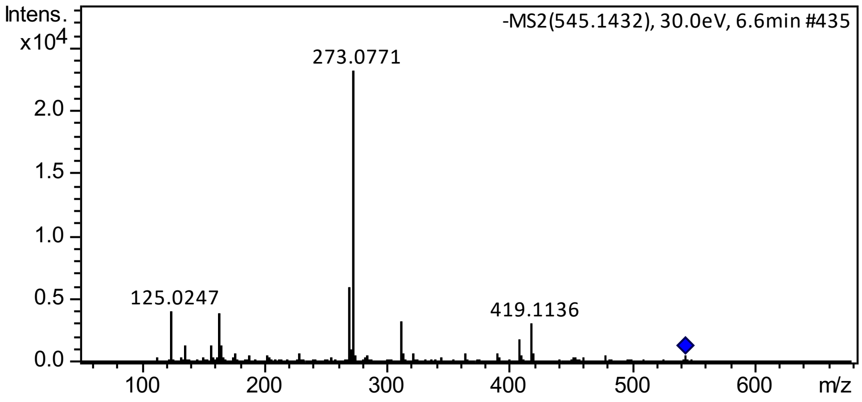

m/z	I %
273.0771	100.0
271.0614	25.7
125.0247	17.4
164.0115	16.6
274.0805	15.5
312.0643	14.0
419.1136	13.1
313.0701	9.1
409.0940	7.4
165.0178	5.4
157.0872	5.3

Compound 23 - Orientin

UHPLC (Rt min)	Measured *m/z*	Ion Formula [M-H]⁻	*m/z*	Error (ppm)	eV(MS⁻)
6.80	447.0947	$C_{21}H_{19}O_{11}$	447.0933	3.1	30

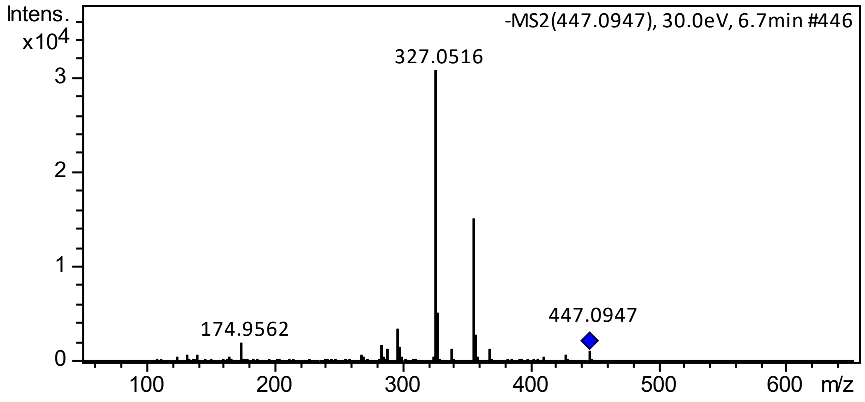

m/z	I %
327.0516	100.0
357.0618	48.9
328.0543	16.8
297.0401	10.9
358.0655	9.2
174.9562	6.1
285.0408	5.3
299.0562	4.9
339.0518	3.9
289.0727	3.9
369.0613	3.8

Compound 24 - Kaempferol-hexose-deoxyhexose

UHPLC (Rt min)	Measured *m/z*	Ion Formula $[M-H]^-$	*m/z*	Error (ppm)	eV(MS^-)
6.80	593.1514	$C_{27}H_{29}O_{15}$	593.1512	-0.3	20

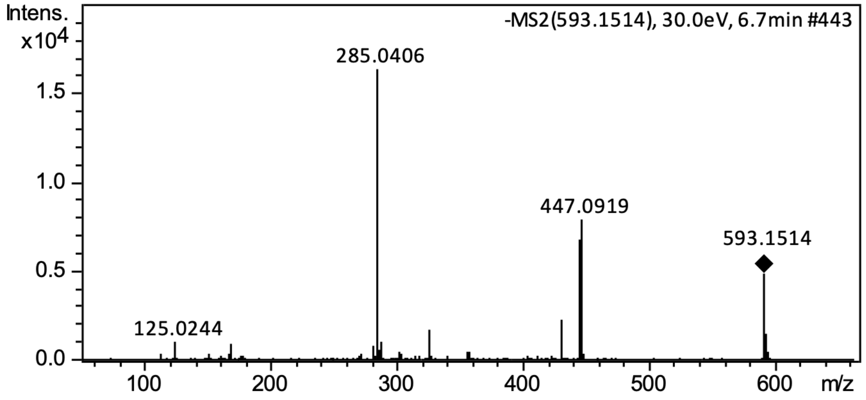

m/z	I %
285.0406	100.0
447.0919	48.1
446.0854	41.3
593.1514	29.4
431.0997	13.5
286.0443	12.9
327.0529	10.3
448.0972	9.7
594.1548	8.8
125.0244	6.2
289.0725	5.9

Compound 25 - Myricetin-pentose(II)

UHPLC (Rt min)	Measured *m/z*	Ion Formula $[M-H]^-$	*m/z*	Error (ppm)	eV(MS^-)
6.90	449.0754	$C_{20}H_{17}O_{12}$	449.0725	6.5	20

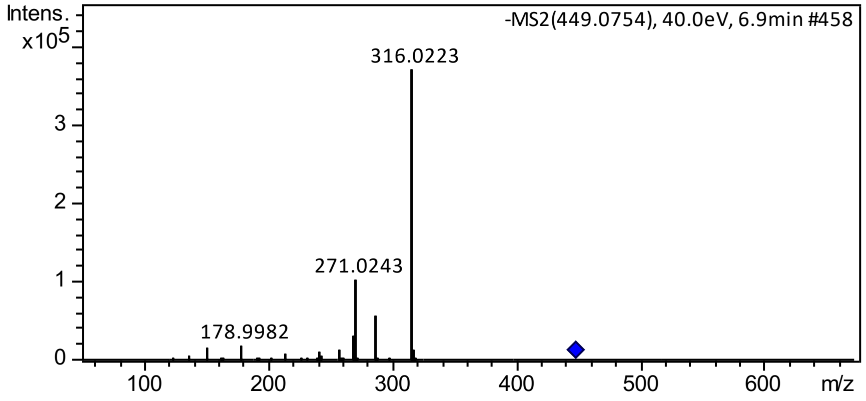

m/z	I %
316.0223	100.0
271.0243	27.2
317.0272	22.7
287.0193	15.4
270.0166	8.3
178.9982	5.0
288.0251	4.5
151.0034	4.2
272.0280	4.1
318.0296	3.7
259.0242	3.3
242.0217	3.1
214.0267	1.8
243.0285	1.5
137.0243	1.3

Compound 26 - Catechin gallate

UHPLC (Rt min)	Measured *m/z*	Ion Formula [M-H]$^-$	*m/z*	Error (ppm)	eV(MS$^-$)
7.00	441.0832	$C_{22}H_{17}O_{10}$	441.0827	1.1	40

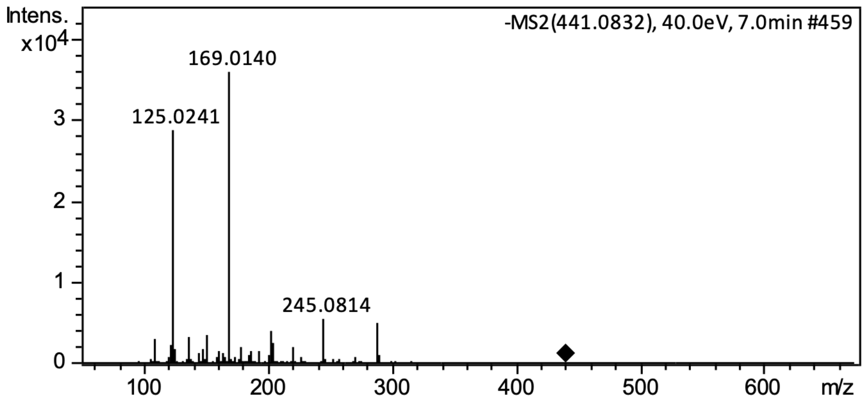

m/z	I %
169.0140	100.0
125.0241	79.8
245.0814	15.4
289.0709	13.6
124.0163	12.8
203.0715	11.1
137.0242	9.3
151.0396	9.3
109.0288	8.1
170.0167	7.6
205.0504	7.2
123.0452	6.0
179.0348	5.7

Compound 27 – Myricitrin-215

UHPLC (Rt min)	Measured *m/z*	Ion Formula [M-H]$^-$	*m/z*	Error (ppm)	eV(MS$^-$)
7.4	531.0753	$C_{31}H_{15}O_9$	531.0722	5.8	20

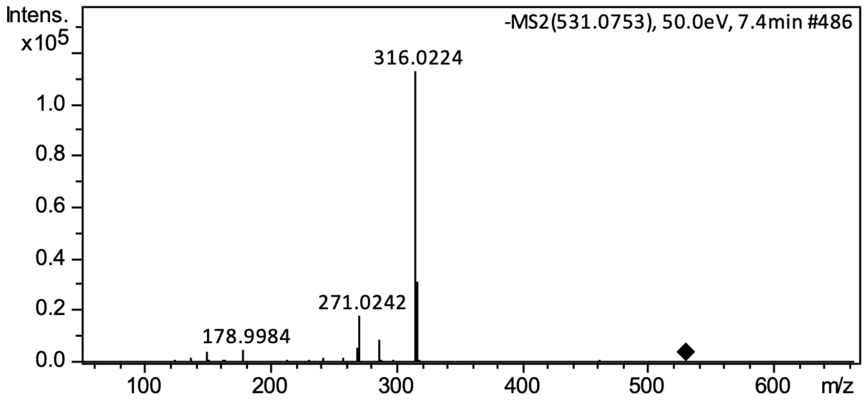

m/z	I %
316.0224	100.0
317.0278	27.9
271.0242	15.9
287.0199	7.9
270.0170	4.8
318.0306	4.6
178.9984	4.3
151.0034	3.5
288.0260	3.1
272.0278	2.4
137.0238	1.4

Compound 28 - Myricitrin

UHPLC (Rt min)	Measured *m/z*	Ion Formula [M-H]⁻	*m/z*	Error (ppm)	eV(MS⁻)
7.3	463.0874	$C_{21}H_{19}O_{12}$	463.0882	-1.7	40

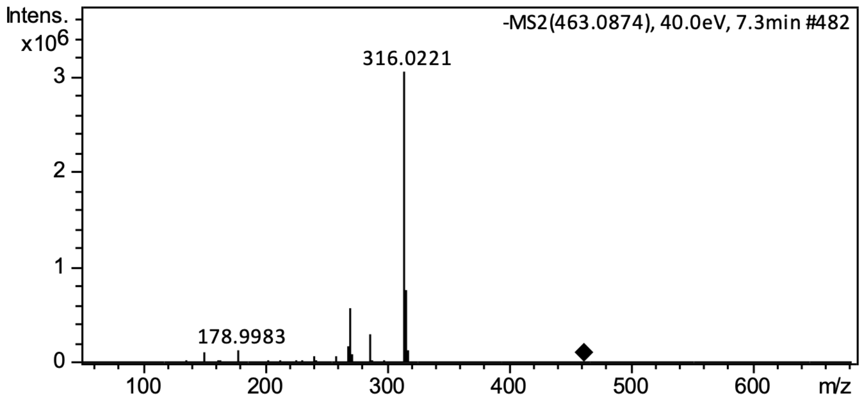

m/z	I %
316.0221	100.0
317.0275	25.1
271.0243	18.2
287.0194	9.8
270.0166	5.8
178.9983	4.1
318.0297	3.9
288.0253	3.5
151.0033	3.1
272.0277	2.8

Compound 29 - Quercetin-441

UHPLC (Rt min)	Measured *m/z*	Ion Formula [M-H]⁻	*m/z*	Error (ppm)	eV(MS⁻)
7.5	741.1896	$C_{32}H_{37}O_{20}$	741.1883	1.75	50

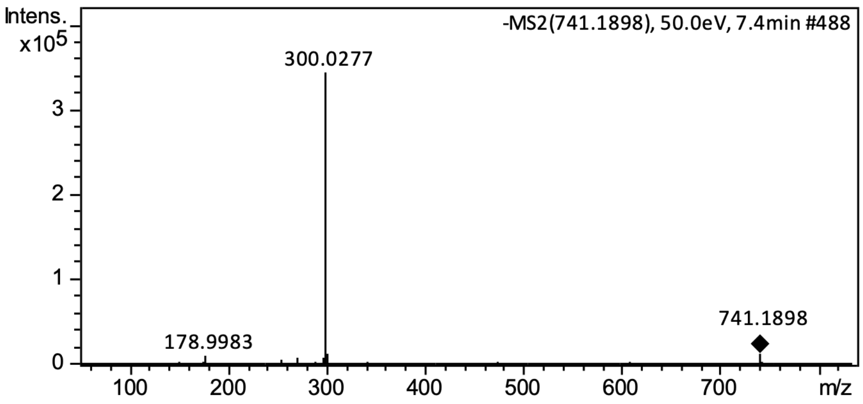

m/z	I %
300.0277	100.0
301.0327	24.9
741.1898	3.6
302.0352	3.6
178.9983	2.8
271.0248	2.2
299.0194	2.0
742.1918	1.4
255.0295	1.1
151.0034	1.0
272.0313	0.7
289.0722	0.5
303.0392	0.5

Compound 30 - Isovitexin

UHPLC (Rt min)	Measured *m/z*	Ion Formula [M-H]$^-$	*m/z*	Error (ppm)	eV(MS$^-$)
7.6	431.0998	$C_{21}H_{19}O_{10}$	431.0984	3.2	30

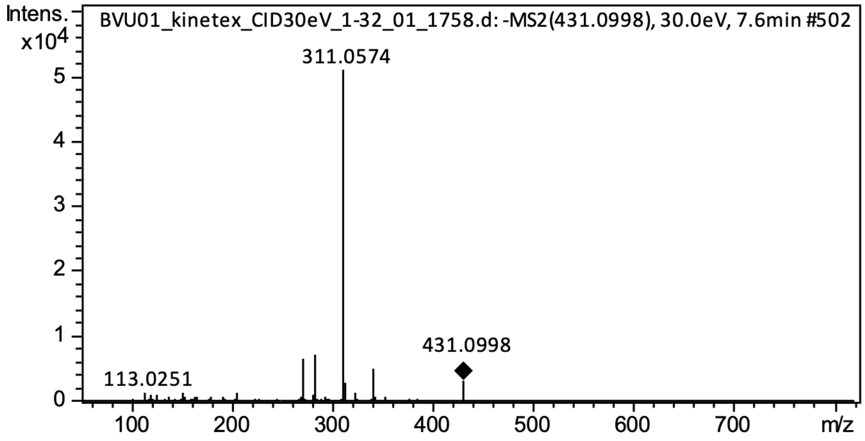

m/z	I %
311.0574	100.0
312.0606	19.5
283.0618	14.1
271.0625	12.5
341.0683	9.6
431.0998	6.2
313.0688	5.4
272.0664	2.4
284.0663	2.4
323.0563	2.4
113.0251	2.4
205.1246	2.3
151.0037	2.2

Compound 31 - Peltatoside

UHPLC (Rt min)	Measured *m/z*	Ion Formula $[M-H]^-$	*m/z*	Error (ppm)	$eV(MS^-)$
7.7	595.1316	$C_{26}H_{27}O_{16}$	595.1305	1.84	20

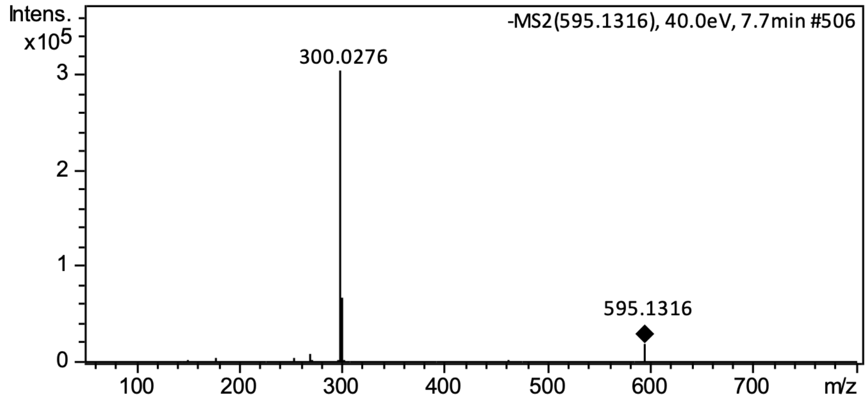

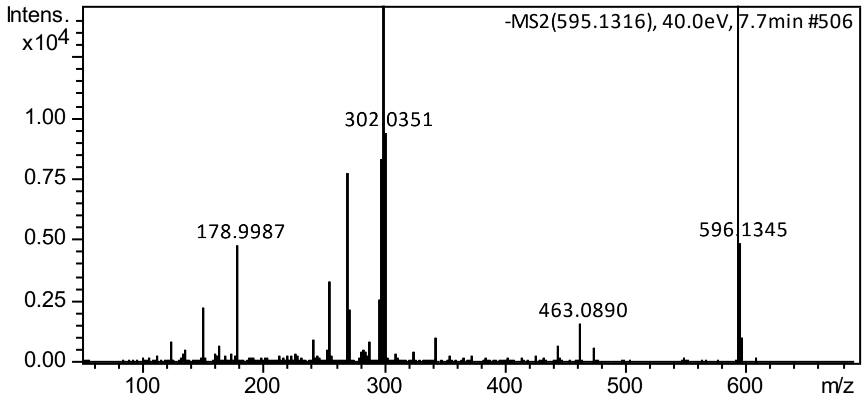

m/z	I %
300.0276	100.0
301.0322	22.0
595.1316	6.1
302.0351	3.1
299.0199	2.7
271.0244	2.5
596.1345	1.6
178.9987	1.6
255.0296	1.1
270.0169	0.9
298.0120	0.8
272.0309	0.7
151.0035	0.7
463.0890	0.5

Compound 32 - Kaempferol-deoxyhexose-deoxyhexose-hexose

UHPLC (Rt min)	Measured *m/z*	Ion Formula $[M-H]^-$	*m/z*	Error (ppm)	$eV(MS^-)$
7.8	739.2126	$C_{33}H_{39}O_{19}$	739.2091	4.7	50

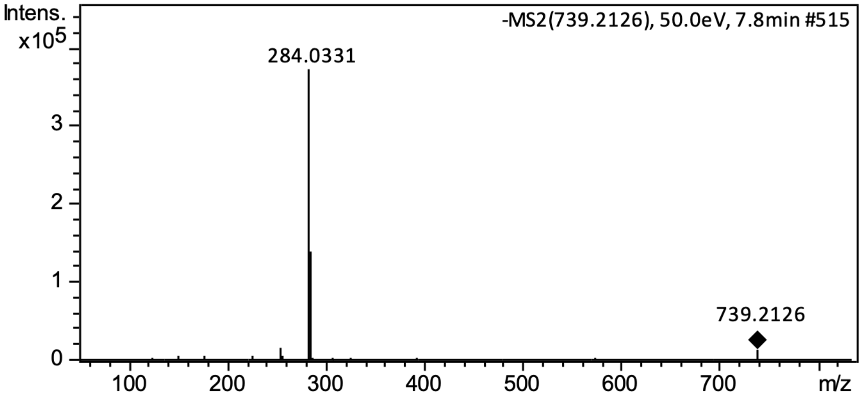

m/z	I %
284.0331	100.0
285.0390	37.0
286.0424	5.5
255.0303	4.0
739.2126	3.1
227.0350	1.7
256.0369	1.7
283.0252	1.6
178.9986	1.5
740.2152	1.3
151.0038	1.2
257.0447	1.1

Compound 33 - Quercetin-309

UHPLC (Rt min)	Measured *m/z*	Ion Formula $[M-H]^-$	*m/z*	Error (ppm)	$eV(MS^-)$
8.0	609.1468	$C_{27}H_{29}O_{16}$	609.1450	3.0	35

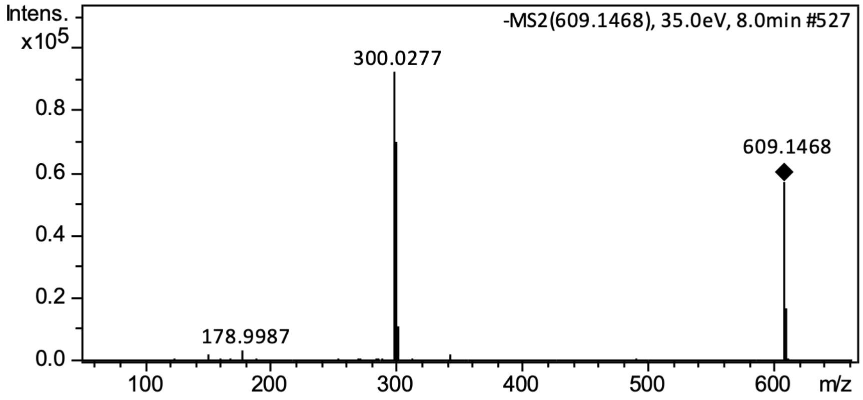

m/z	I %
300.0277	100.0
301.0348	75.7
609.1468	61.4
610.1501	17.9
302.0379	11.6
611.1527	4.5
178.9987	3.3
151.0038	2.3
343.0459	2.0
303.0405	1.3

Compound 34 - Quercetin-hexose

UHPLC (Rt min)	Measured *m/z*	Ion Formula [M-H]-	*m/z*	Error (ppm)	eV(MS-)
7.80	463.0886	$C_{21}H_{19}O_{12}$	463.0882	0.8	30

•

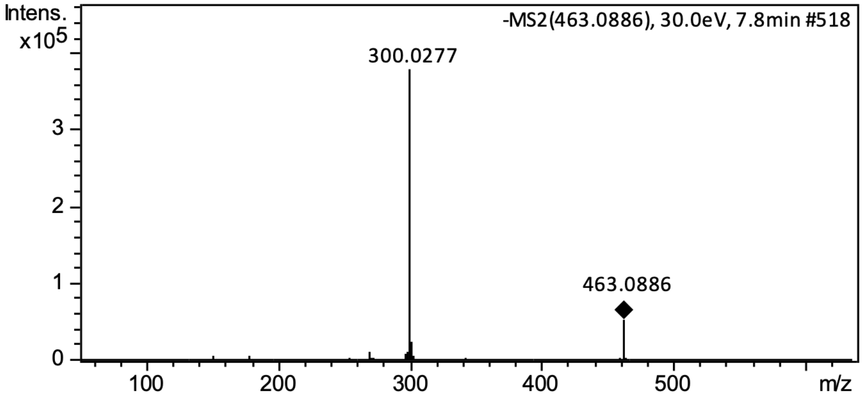

m/z	I %
300.0277	100.0
301.0342	44.7
463.0886	13.7
302.0368	6.1
299.0194	3.0
271.0249	2.9
464.0917	2.6
298.0122	2.0
270.0175	1.8
178.9985	1.6
151.0038	1.4

Compound 35 - Isorhamnetin-471

UHPLC (Rt min)	Measured *m/z*	Ion Formula [M-H]-	*m/z*	Error (ppm)	eV(MS-)
7.95	785.2153	$C_{34}H_{31}O_{21}$	785.2146	0.9	50

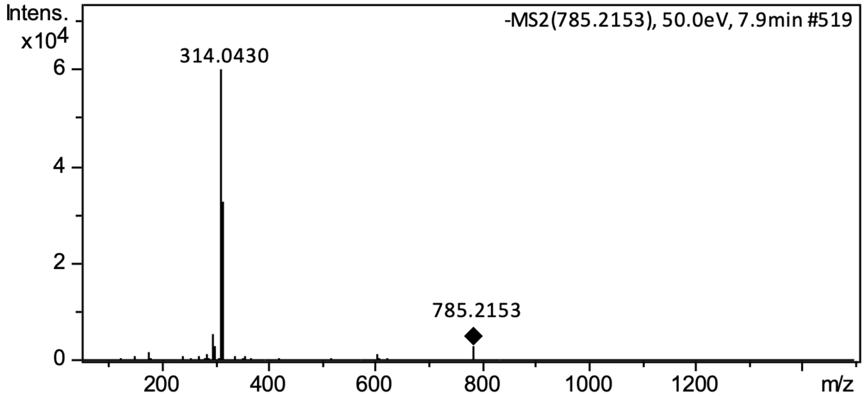

m/z	I %
314.0430	100.0
315.0496	54.3
299.0199	9.3
316.0535	8.6
313.0352	5.4
300.0247	4.8
785.2153	4.6
178.9979	2.9
605.1514	2.0
786.2172	1.9
286.0480	1.8

Compound 36 - Kaempferol-308

UHPLC (Rt min)	Measured *m/z*	Ion Formula [M-H]$^-$	*m/z*	Error (ppm)	eV(MS$^-$)
8.1	593.1536	$C_{27}H_{29}O_{15}$	593.1512	4.0	50

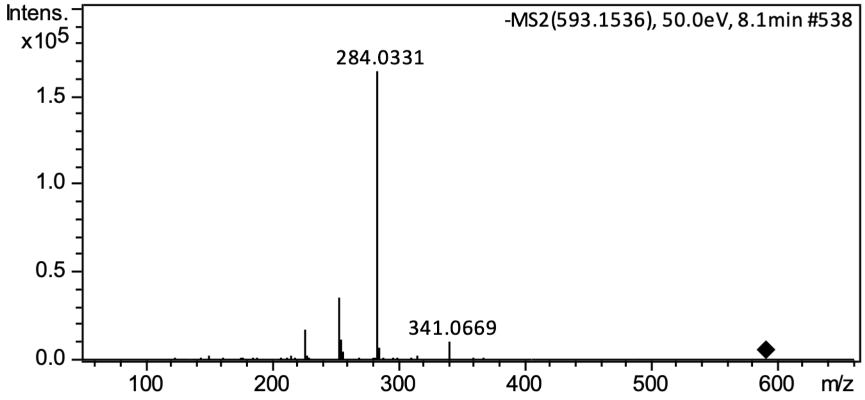

m/z	I %
284.0331	100.0
285.0388	31.8
255.0303	21.4
227.0351	10.2
256.0361	6.8
341.0669	6.0
286.0418	4.3
257.0440	2.5
229.0499	1.6
228.0385	1.4
151.0040	1.4

Compound 37 - NCGC00384841

UHPLC (Rt min)	Measured *m/z*	Ion Formula [M-H]$^-$	*m/z*	Error (ppm)	eV(MS$^-$)
8.1	539.2110	$C_{26}H_{35}O_{12}$	539.2134	-4.5	30

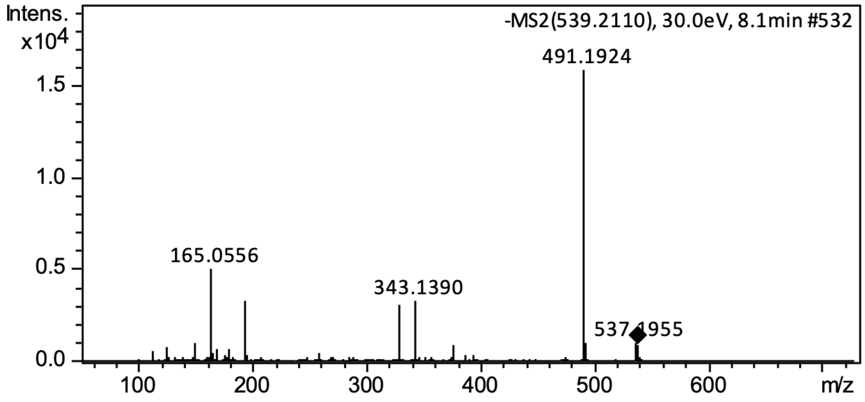

m/z	I %
491.1924	100.0
165.0556	32.1
492.1962	24.9
195.0656	21.0
343.1390	20.6
329.1393	19.7
537.1955	6.2
493.1980	6.0
150.0326	6.0
377.1621	5.9
539.2110	5.8

Compound 38 - Azelaic Acid

UHPLC (Rt min)	Measured *m/z*	Ion Formula [M-H]$^-$	*m/z*	Error (ppm)	eV(MS$^-$)
8.2	187.0969	$C_9H_{15}O_4$	187.0976	-3.7	20

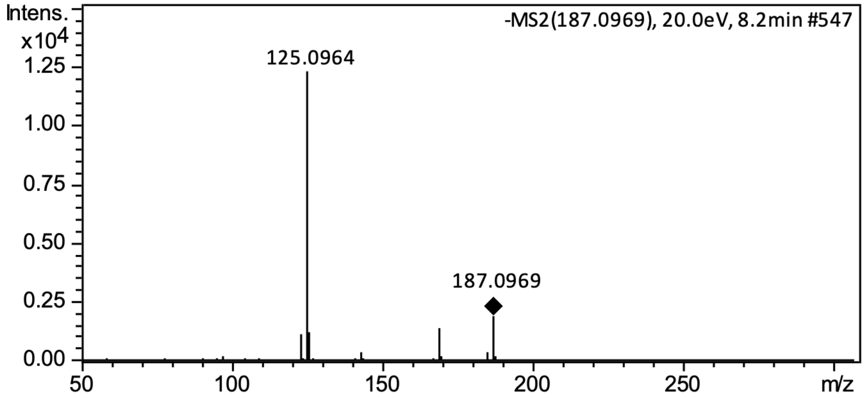

m/z	I %
125.0964	100.0
187.0969	15.0
169.0860	10.8
126.1001	9.8
123.0810	9.1
143.1079	3.0
185.1179	2.5

Compound 39 - Vitexin

UHPLC (Rt min)	Measured *m/z*	Ion Formula [M-H]$^-$	*m/z*	Error (ppm)	eV(MS$^-$)
8.3	431.0985	$C_{21}H_{19}O_{10}$	431.0984	0.2	40

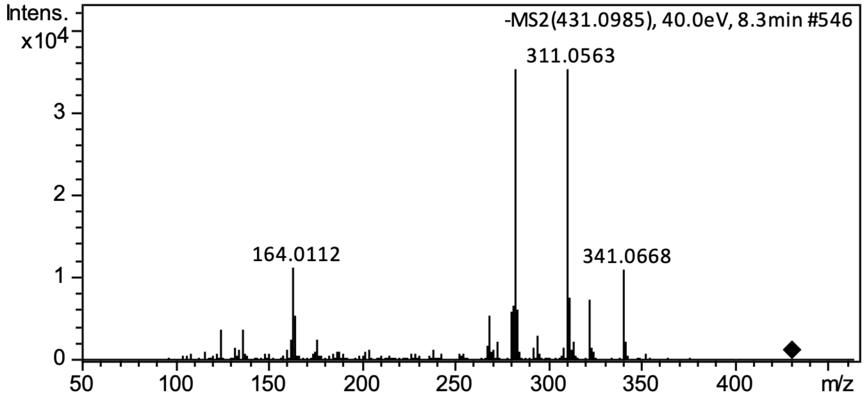

m/z	I %
311.0563	100.0
283.0612	99.9
164.0112	31.7
341.0668	31.0
312.0601	21.4
323.0561	20.8
282.0527	18.8
284.0640	17.2
281.0459	16.5
165.0184	15.1
269.0454	15.0

Compound 40 - Metilquercetin-455

UHPLC (Rt min)	Measured *m/z*	Ion Formula $[M-H]^-$	*m/z*	Error (ppm)	$eV(MS^-)$
8.50	769.2211	$C_{34}H_{41}O_{20}$	769.2197	1.8	50

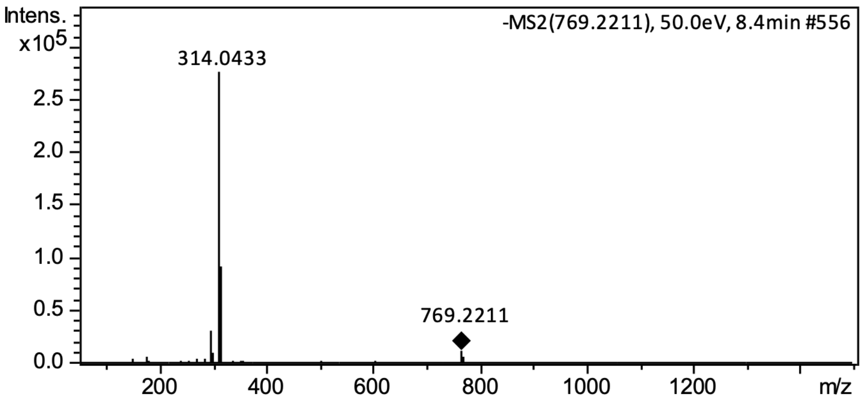

m/z	I %
314.0433	100.0
315.0488	33.3
299.0198	10.8
316.0522	4.7
769.2211	4.0
300.0254	3.5
178.9982	2.3
313.0352	2.1
770.2237	1.8
271.0247	1.6
151.0034	1.2

Compound 41 - Avicularin

•

UHPLC (Rt min)	Measured *m/z*	Ion Formula $[M-H]^-$	*m/z*	Error (ppm)	$eV(MS^-)$
8.50	433.0774	$C_{20}H_{17}O_{11}$	433.0776	-0.5	40

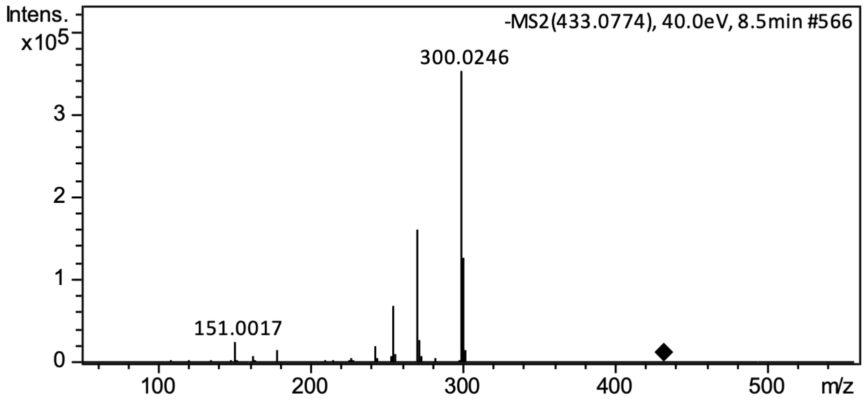

m/z	I %
300.0246	100.0
271.0220	45.2
301.0305	36.0
255.0270	19.3
272.0265	7.7
151.0017	7.1

Compound 42 - Kaempferol-308 II

UHPLC (Rt min)	Measured *m/z*	Ion Formula $[M-H]^-$	*m/z*	Error (ppm)	eV (MS^-)
8.50	593.1537	$C_{27}H_{29}O_{15}$	593.1512	4.2	45

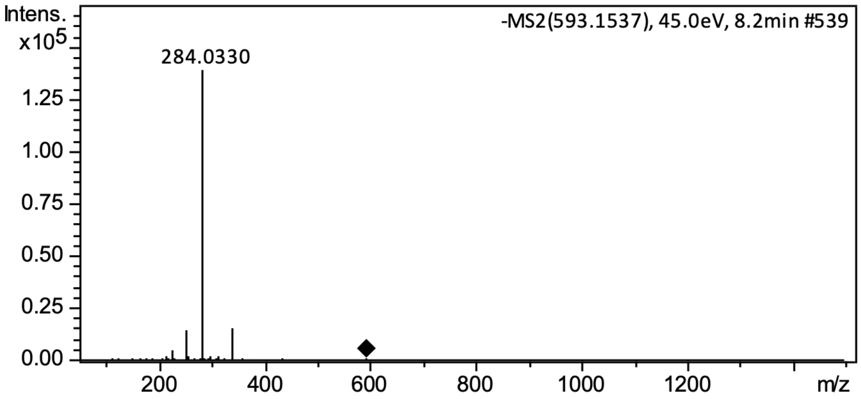

m/z	I %
284.0330	100.0
285.0389	34.0
341.0667	11.0
255.0300	10.3
286.0414	5.1
256.0363	3.6
227.0353	3.5
301.0358	1.5

Compound 43 - Quercitrin

UHPLC (Rt min)	Measured *m/z*	Ion Formula $[M-H]^-$	*m/z*	Error (ppm)	eV (MS^-)
9.10	447.0907	$C_{21}H_{19}O_{11}$	447.0933	-5.8	40

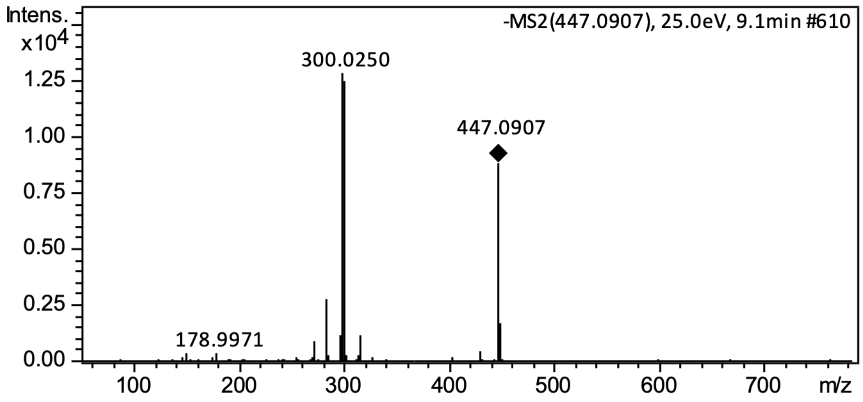

m/z	I %
300.0250	100.0
301.0328	96.9
447.0907	68.4
284.0302	21.6
448.0942	15.9
449.0705	13.2
302.0360	12.6
285.0373	11.5
151.0026	2.6
178.9971	2.7

Compound 44 - (epi)afzelechin-(epi)catechin(ii)

UHPLC (Rt min)	Measured *m/z*	Ion Formula [M-H]$^-$	*m/z*	Error (ppm)	eV(MS$^-$)
9.20	561.1430	$C_{30}H_{25}O_{11}$	561.1402	4.9	30

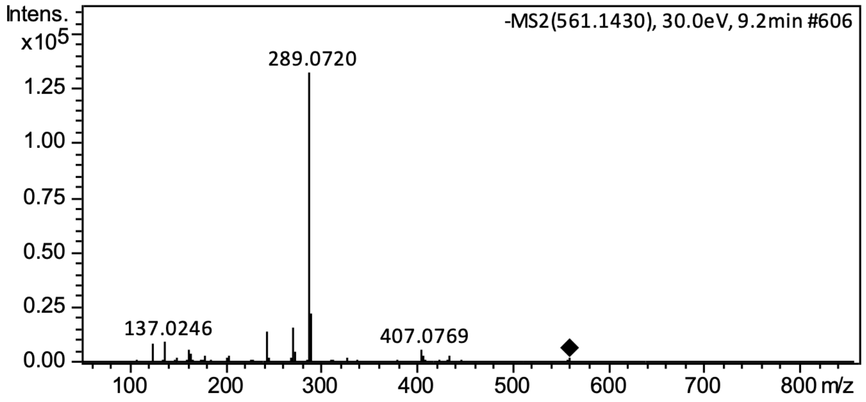

m/z	I %
289.0720	100.0
290.0755	16.8
271.0615	11.9
245.0823	10.1
137.0246	7.2
125.0243	6.3
164.0114	4.1
407.0769	4.0
273.0768	3.7
165.0184	2.5
291.0772	2.3
409.0923	2.2
179.0351	2.1

Compound 45 - Isorhamnetin-hexose

UHPLC (Rt min)	Measured *m/z*	Ion Formula [M-H]$^-$	*m/z*	Error (ppm)	eV(MS$^-$)
9.9	477.1033	$C_{22}H_{21}O_{12}$	477.1038	-1.1	40

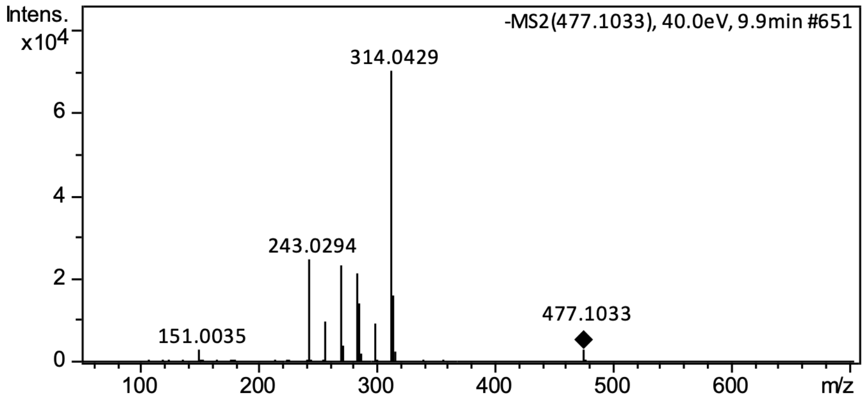

m/z	I %
314.0429	100.0
243.0294	34.9
271.0243	32.8
285.0401	29.9
315.0479	22.8
286.0473	20.0
257.0451	13.8
299.0190	13.1
271.0609	12.7
300.0260	5.6
272.0276	5.3
244.0324	4.6

Compound 46 - Naringenin 7-O-glucoside

UHPLC (Rt min)	Measured *m/z*	Ion Formula $[M-H]^-$	*m/z*	Error (ppm)	$eV(MS^-)$
10.05	433.1160	$C_{21}H_{21}O_{10}$	433.1140	4.6	35

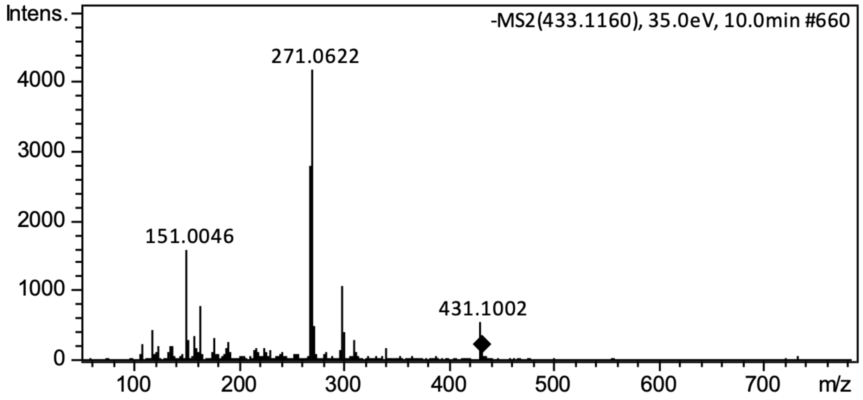

m/z	I %
271.0622	100.0
268.0391	66.7
151.0046	37.6
300.0283	25.8
269.0435	23.4
165.0203	18.8
431.1002	13.0
272.0661	11.5
119.0514	10.1
301.0331	9.6

Compound 47 - Kaempferol-131

UHPLC (Rt min)	Measured *m/z*	Ion Formula $[M-H]^-$	*m/z*	Error (ppm)	$eV(MS^-)$
10.3	415.1946	$C_{20}H_{31}O_9$	415.1974	-6.7	30

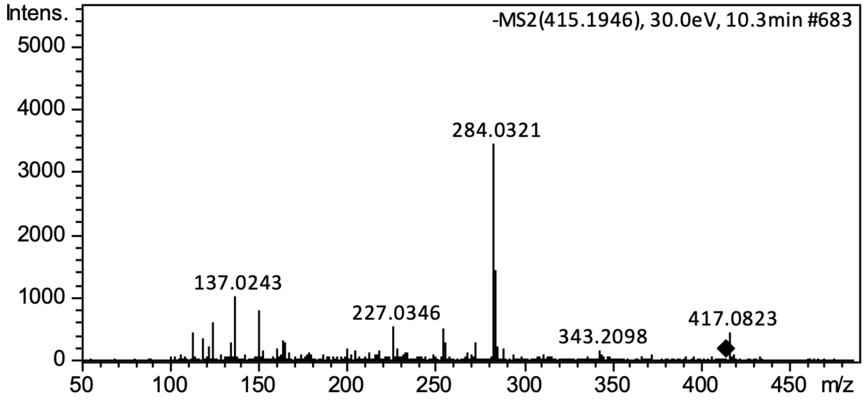

m/z	I %
284.0321	100.0
285.0399	41.7
137.0243	29.8
151.0410	22.9
125.0246	17.4
227.0346	15.3
255.0297	14.4
417.0823	13.4
113.0242	13.4
137.0965	11.7
119.0356	10.0
164.0108	9.6

Compound 48 - 3', 4', 7, 8 -Tetrahydroxyflavone

UHPLC (Rt min)	Measured *m/z*	Ion Formula [M-H]$^-$	*m/z*	Error (ppm)	eV(MS$^-$)
10.37	287.0561	$C_{15}H_{11}O_6$	287.0561	0.0	20

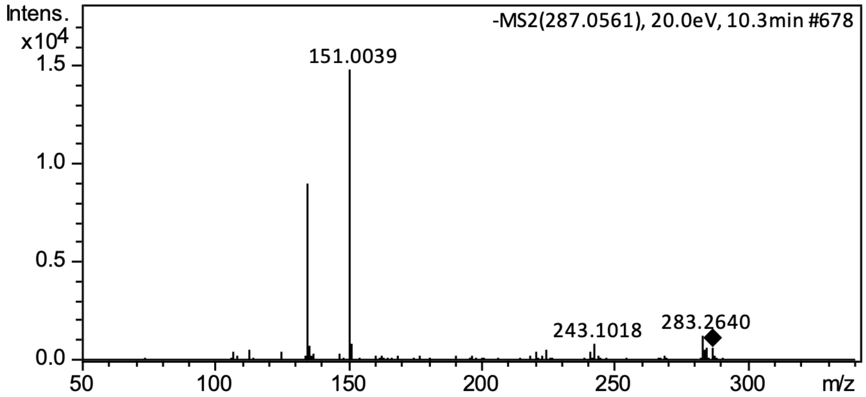

m/z	I %
151.0039	100.0
135.0454	60.9
283.2640	8.4
243.1018	5.4
152.0075	5.3
136.0492	4.8
285.1328	4.4
287.0561	4.0

Compound 49 - Kaempferol-214

UHPLC (Rt min)	Measured *m/z*	Ion Formula [M-H]$^-$	*m/z*	Error (ppm)	eV(MS$^-$)
10.5	499.0860	$C_{24}H_{19}O_{12}$	499.0882	-4.4	40

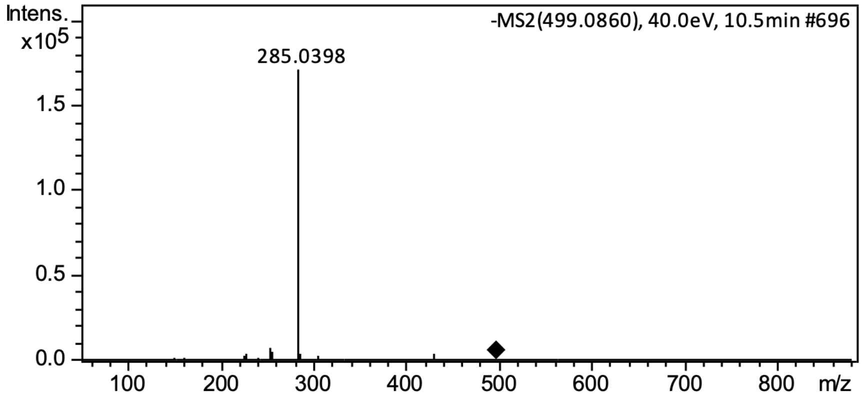

m/z	I %
285.0398	100.0
284.0323	71.7
286.0431	15.1
255.0295	4.2
257.0447	2.5
431.0984	2.4
287.0448	2.1
229.0502	2.1
256.0355	2.0
227.0350	1.6
307.0220	1.5

Compound 50 - Kaempferide-116

UHPLC (Rt min)	Measured *m/z*	Ion Formula [M-H]$^-$	*m/z*	Error (ppm)	eV(MS$^-$)
10.7	417.2126	$C_{20}H_{33}O_9$	417.2119	1.7	40

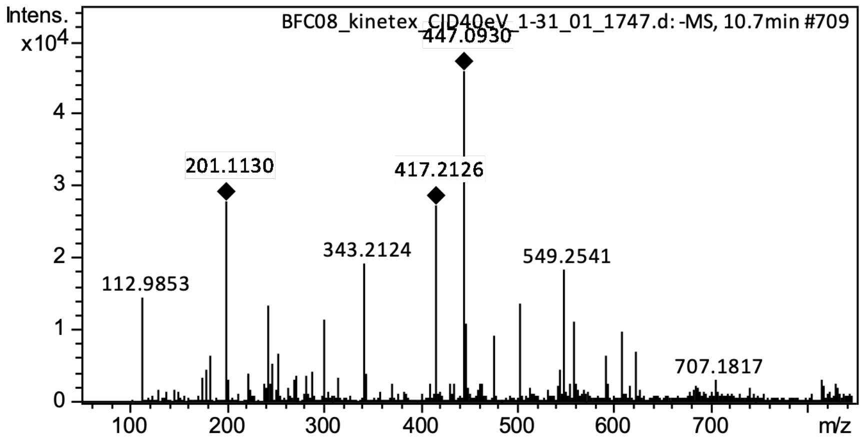

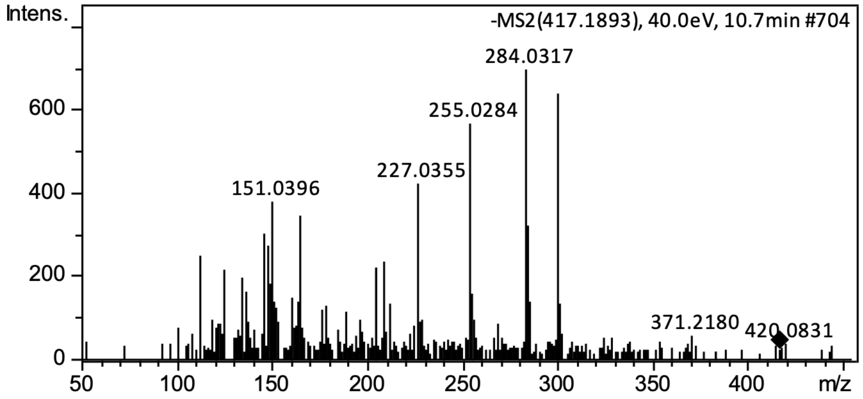

m/z	I % (40eV)
284.0317	100.0
301.0367	91.5
255.0284	81.0
227.0355	60.9
151.0396	54.4
165.0541	49.5
151.0038	48.5
285.0393	46.2
146.9612	43.1
149.0238	39.5

Compound 51 - Quercetin-313

UHPLC (Rt min)	Measured *m/z*	Ion Formula [M-H]$^-$	*m/z*	Error (ppm)	eV(MS$^-$)
10.80	475.0874	$C_{22}H_{19}O_{12}$	475.0882	-1.7	20

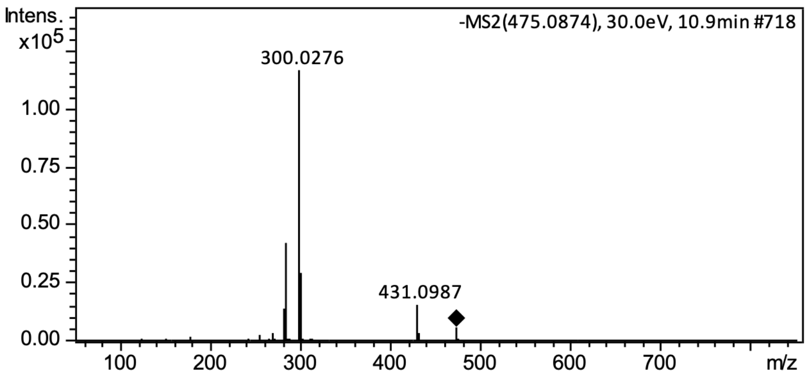

m/z	I %
300.0276	100.0
285.0402	35.8
301.0327	24.8
431.0987	13.5
284.0323	12.0
475.0874	4.7
286.0434	4.6
302.0348	3.6
271.0245	3.0
432.1028	2.5
255.0295	1.8
178.9986	1.1

Compound 52 - Trihydroxyflavone-dimetil-161

UHPLC (Rt min)	Measured *m/z*	Ion Formula $[M-H]^-$	*m/z*	Error (ppm)	eV(MS^-)
11.7	461.1450	$C_{23}H_{25}O_{10}$	461.1453	-0.7	20

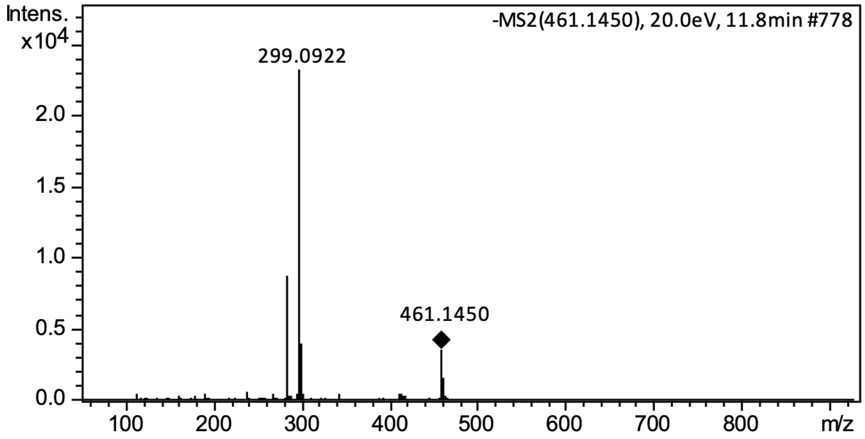

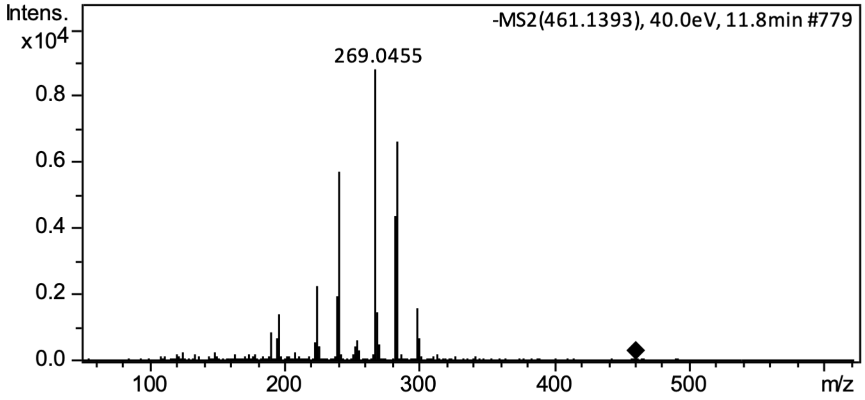

m/z	I % (40 eV)
269.0455	100.0
284.0685	75.0
241.0506	64.9
283.0610	49.5
225.0552	25.7
240.0419	22.3
268.0373	18.1
300.0272	17.8
270.0491	16.7
197.0603	15.9
242.0534	13.4
191.0349	9.7

Compound 53 - 7,4'-Dimethoxy-5-hydroxyflavone-203

UHPLC (Rt min)	Measured *m/z*	Ion Formula [M-H]$^-$	*m/z*	Error (ppm)	eV(MS$^-$)
11.7	503.1557	$C_{25}H_{27}O_{11}$	503.1547	1.9	35

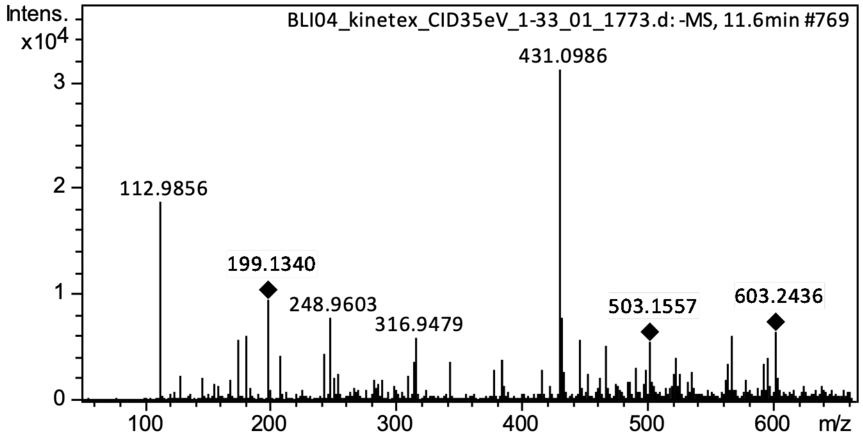

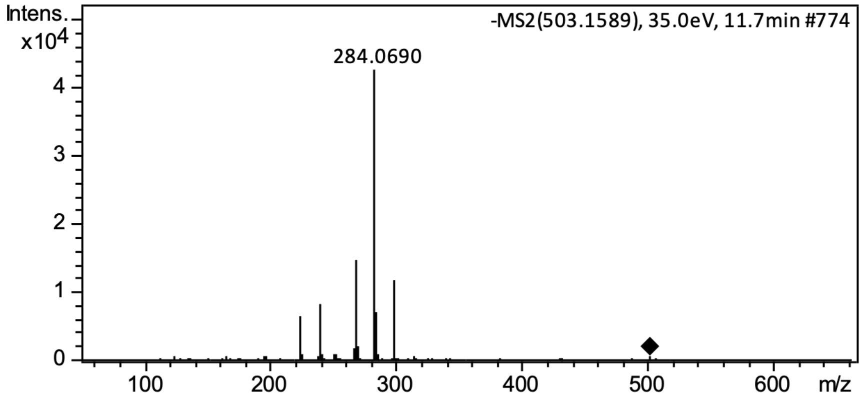

m/z	I %
284.0690	100.0
283.0617	34.5
269.0459	34.2
299.0925	27.2
241.0506	19.6
285.0724	16.2
225.0559	14.9
240.0792	6.4
285.0408	4.7
270.0492	4.7
240.0431	4.3

Compound 54 - Naringenin Falcone

UHPLC (Rt min)	Measured *m/z*	Ion Formula [M-H]$^-$	*m/z*	Error (ppm)	eV(MS$^-$)
12.1	271.0614	$C_{15}H_{11}O_5$	271.0612	0.7	30

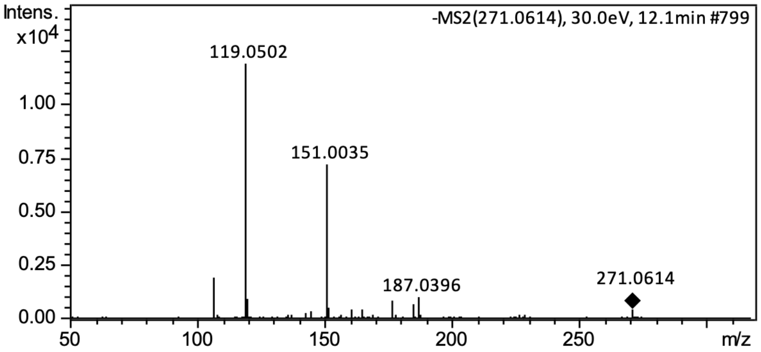

m/z	I %
119.0502	100.0
151.0035	60.5
107.0132	16.0
187.0396	8.7
120.0538	7.9
177.0189	6.7
185.0604	5.5
152.0068	3.9
161.0602	3.8
165.0194	3.2
271.0614	3.1
145.0297	2.6

Compound 55 - Bauhiniastatin2

UHPLC (Rt min)	Measured *m/z*	Ion Formula [M-H]$^-$	*m/z*	Error (ppm)	eV(MS$^-$)
14.60	299.1417	$C_{17}H_{15}O_5$	299.1401	5.3	30

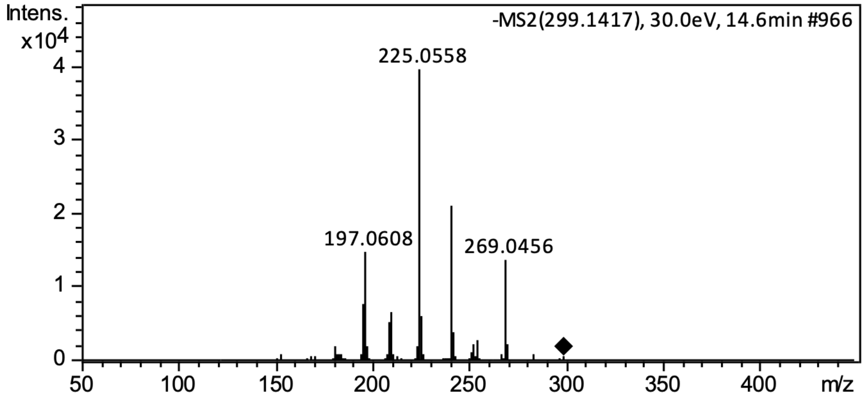

m/z	I %
225.0556	100.0
197.0605	83.1
241.0505	75.3
210.0320	36.1
196.0525	27.2
269.0453	25.6
209.0605	19.7
226.0587	15.0
181.0657	12.0
195.0446	11.7
242.0536	11.0
198.0640	10.5
167.0499	9.2
224.0476	8.5
